# High cortisol in 5-year-old children causes loss of DNA methylation in SINE retrotransposons: a possible role for ZNF263 in stress-related diseases

**DOI:** 10.1186/s13148-015-0123-z

**Published:** 2015-09-04

**Authors:** Daniel Nätt, Ingela Johansson, Tomas Faresjö, Johnny Ludvigsson, Annika Thorsell

**Affiliations:** Department of Clinical and Experimental Medicine, Division of Cell Biology, Linkoping University, Linkoping, 58183 Sweden; Department of Clinical and Experimental Medicine, Division of Pediatrics, Linkoping University, Linkoping, 58183 Sweden; Department of Medicine and Health Sciences, Community Medicine/General Practice, Linkoping University, Linkoping, 58183 Sweden

**Keywords:** Stress, DNA methylation, ZNF263, Children, Retrotransposon, Cortisol, Transcription factor, EGR1, Blood, Hair

## Abstract

**Background:**

Childhood stress leads to increased risk of many adult diseases, such as major depression and cardiovascular disease. Studies show that adults with experienced childhood stress have specific epigenetic changes, but to understand the pathways that lead to disease, we also need to study the epigenetic link prospectively in children.

**Results:**

Here, we studied a homogenous group of 48 5-year-old children. By combining hair cortisol measurements (a well-documented biomarker for chronic stress), with whole-genome DNA-methylation sequencing, we show that high cortisol associates with a genome-wide decrease in DNA methylation and targets short interspersed nuclear elements (SINEs; a type of retrotransposon) and genes important for calcium transport: phenomena commonly affected in stress-related diseases and in biological aging. More importantly, we identify a zinc-finger transcription factor, ZNF263, whose binding sites where highly overrepresented in regions experiencing methylation loss. This type of zinc-finger protein has previously shown to be involved in the defense against retrotransposons.

**Conclusions:**

Our results show that stress in preschool children leads to changes in DNA methylation similar to those seen in biological aging. We suggest that this may affect future disease susceptibility by alterations in the epigenetic mechanisms that keep retrotransposons dormant. Future treatments for stress- and age-related diseases may therefore seek to target zinc-finger proteins that epigenetically control retrotransposon reactivation, such as ZNF263.

**Electronic supplementary material:**

The online version of this article (doi:10.1186/s13148-015-0123-z) contains supplementary material, which is available to authorized users.

## Background

Large-scale cohort data show that individuals experiencing domestic violence, neglect, and abuse during childhood have a higher incidence of, for instance, adult neuropsychiatric problems [1], diabetes [2], and cardiovascular disease [3]. The broad negative effects of early life adversity may even lead to accelerated aging [4] and premature death [5].

Individuals who experience childhood abuse show a hyperactive hypothalamic-pituitary-adrenal (HPA) axis as adults, which is particularly exaggerated in neuropsychiatric disorders, such as major depression [6]. Dysfunction of the HPA axis has also been described in a number of other psychiatric disorders [7], as well as in cardiovascular disease [8], and some metabolic disorders [9]. Measuring cortisol, the end product of the HPA axis, may therefore be an appropriate biomarker when studying the development of these diseases. However, the spatiotemporal signature of cortisol in blood is highly fluctuating, depending on for example diurnal rhythm and physical activity, making it a non-optimal biomarker for chronic stress [10]. We, and others, have recently shown that measuring cortisol in hair samples gives an average measure of cortisol release for a period up to several months [10–12]. These levels also correlate with experienced life stressors and stress-related disorders, making it a more stable biomarker for chronic stress than blood cortisol.

In animal models, chronic stress changes the expression of key enzymes involved in epigenetic regulation, like DNA methyltransferases [13] and histone deacetylases [14]. There is also plenty of evidence showing how chronic stress leads to epigenetic changes in, for example, DNA methylation and histone acetylation, which give rise to disease-like phenotypes [15]. In humans, it is therefore thought that epigenetic mechanisms may play an important role in the detrimental health effects seen in adults with a history of childhood adversity. A few genome-wide studies have indeed reported such associations [16, 17]. Using a candidate gene approach, it has also been shown that childhood abuse is associated with a change in DNA methylation at the glucocorticoid receptor gene (*NR3C1*), an important regulator of the HPA axis [18]. More specifically, this change in DNA methylation targets a binding site of the early growth response 1 (EGR1, also known as NGFI-A, Krox-24, Zif268, Znf225, ZENK), an evolutionary conserved transcription factor that is activated by acute stress [19, 20].

While there have been several attempts to investigate how stress exposure during childhood affects DNA methylation in adult humans, there are currently no published studies on how the epigenome is impacted by chronic exposure to high cortisol levels in young children. While the retrospective studies do provide possible epigenetic mechanisms associated with a disease, to understand the development of that disease, we must investigate material available prior to disease development. Thus, evaluating healthy individuals prospectively, during childhood, is essential for our understanding of how early adversity may affect a diversity of adult diseases.

In the present study, we therefore investigated how hair cortisol levels correlated with differences in the genome-wide DNA-methylation profiles of 48 healthy 5-year-old children. Our main findings show that high cortisol exposure in these children is generally associated with genome-wide hypomethylation similar to that seen in aging, which targets ZNF263 binding sites, SINE retrotransposons, and mainly affects genes involved in neurodevelopment and calcium transport.

## Results and discussion

### Subjects and samples

**Fig. 1:**
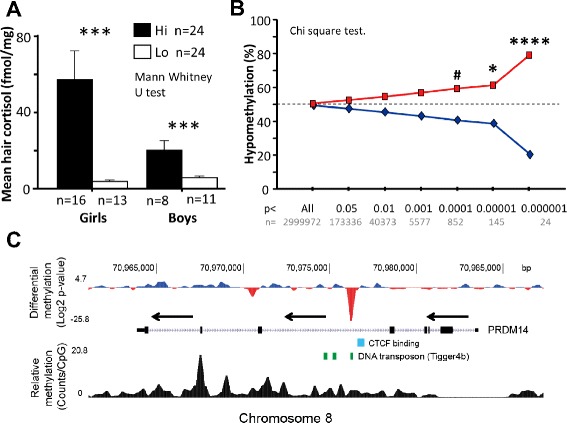
High cortisol was generally associated with DNA hypomethylation. **a** Shows hair cortisol levels in the Hi and Lo groups. Gender was later used as covariate in the whole-genome methylation analysis. **b** The DMRs between Hi and Lo in the whole-genome methylation analysis were primarily hypomethylated in Hi (*red line*) compared to Lo (*blue line*). This relationship diminished with lower whole-genome *p* value of the DMRs, but was still statistically detectable at *p* < 0.0001 when compared to the whole-genome distribution. **c** Genomic view of the most significant DMR between Hi and Lo, located in the fifth intron of *PRDM14* (*red peak*) within a DNA transposon (*green boxes*) and close to a plausible CTCF binding site (*light blue box*). The *top axis* represents the Log2 *p* values of individual 300 bp genomic windows; *red* indicates hypo- and *blue* hypermethylation in Hi (*n* = 24) compared to Lo (*n* = 24) children. The *bottom axis* shows relative methylation, given as mean read counts per window of all children (*n* = 48) divided by number of CpGs in a given window. The *arrows* indicate direction of transcription. Note that the promoter region is completely unmethylated. ****p < 0.0001, ***p < 0.001, *p < 0.05, and #*p* < 0.1. *Error bars* shows standard errors

The 48 children studied here came from a relatively homogenous Southeast Swedish population, previously enrolled in the more comprehensive All Babies in Southeast Sweden (ABIS) project; a cohort initially including 17,000 children born between 1997 and 1999, that were followed prospectively to identify environmental risk factors for complex diseases. Sociodemographic data is presented in Additional file 1: Table S1. Hair samples were taken from the same area close to the scalp. Cortisol levels were then measured using a competitive radioimmunoassay on the flash frozen pulverized hair, and the levels were used to group each child into either a chronically high (Hi) or low (Lo) stress group (Fig. 1a). Since evidence points to a system-wide effect of early life stress, affecting multiple diseases (see “Background” section), we extracted whole blood DNA from each child.

### Stress in children is generally associated with hypomethylation

DNA was used to generate DNA-methylation profiles by whole-genome methylated DNA immunoprecipitation sequencing (MeDIP-seq). In order to search for differentially methylated genomic regions (DMRs) between Hi and Lo, individual sequence reads were counted over 300 bp windows across the human genome. After filtering for low count windows, count data were used in a generalized linear model with gender as a covariate. A summary of the bioinformatics tools used in this paper is presented in Additional file 1: Table S2.

The majority of the most significant DMRs were hypomethylated in the Hi children (Fig. 1b; Additional file 1: Table S3). While this relationship was strongest at lower *p* values (FDR corrected *p* < 0.05 = 10 DMRs, of which 8 were hypo- and 2 hypermethylated), it was statistically detectable down to a significance level of *p* < 0.0001, involving the top 852 DMRs (of which 507 were hypo- and 345 hypermethylated). The strongest DMR was located near an intronic DNA transposon in the *PRDM14* gene (Fig. 1c; for individual methylation profiles, see Additional file 1: Figure S1). Notably, this gene encodes a protein that directly affects TET-mediated demethylation of DNA [21]; hence, it may be involved in generating the genome-wide hypomethylation seen in Hi children. Genome-wide loss of DNA methylation has been seen in many types of diseases such as cancer [22], autoimmune diseases [23], and some neuropsychiatric disorders [24, 25]. There are also accumulating evidence that it may accelerate senescence contributing to age-related disorders such as dementia and cardiovascular disease [26, 27]. That we observe similar events in our methylation profiles indicates that high cortisol exposure early in life may trigger the same pathways.

### Differential methylation not explained by genetic heterogeneity or stress induced bias in cell types

**Fig. 2:**
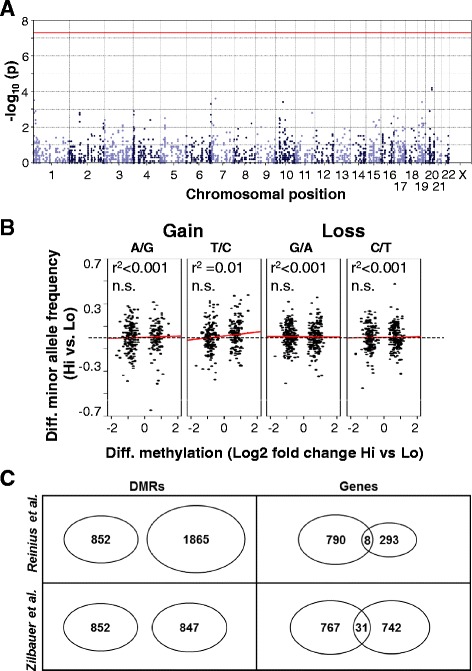
Differentially methylated regions were not affected by genetic variation or variation in blood cell type composition. **a** Manhattan plot of the results from the genetic association study covering 900 bp centered on each *p* < 0.0001 DMR (*n* = 852). *Dots* represent individual SNPs and *red line* the threshold for genome-wide significance. **b** Scatter plots illustrating the correlations between the relative minor allele frequencies of DMR-associated SNPs in Hi compared to Lo, and differential methylation of Hi compared to Lo of the same DMR. If a relationship between allele frequency and methylation level is present, higher relative frequency of alleles that cause gain of methylation sites in hypermethylated DMRs should result in a positive correlation, while the opposite relationship should be expected with alleles causing loss of methylation sites. In hypomethylated DMRs the reverse relationship is expected. **c** Overlap between the DMRs, or their nearest genes, and regions/genes from two independent studies reporting differential methylation between blood leukocytes; Reinius et al. 2012 [29] and Zilbauer et al. 2013 [30]

Two confounding factors may explain the appearance of DMRs in our study: (1) genetic variations between Hi and Lo that affect the number of available methylation sites, and (2) cortisol-induced changes in blood cell composition. To assess the former, we performed a genetic association study between cortisol groups on the *p* < 0.0001 DMRs (*n* = 852) using the sequenced reads acquired for the MeDIP-seq analysis. Immunoprecipitation allows for this type of secondary analysis since it preserves the underlying genetic information which is often lost when using other methylome methods, such as bisulfite sequencing or the popular Infinium HumanMethylation450 BeadChips. None of the detected 2228 single nucleotide polymorphisms (SNPs) was significantly overrepresented in either the Hi or the Lo group (Fig. 2a). Since MeDIP enriches for methylated CpGs, we detected a 3–4 time overrepresentation of C/T, G/A, T/C, and A/G alleles in the DMRs. These alleles constitute the main genetic variation affecting the number of methylation sites in the DMRs, where gain or loss of these sites within either Hi or Lo may explain the methylation differences between the two groups. There was a weak negative correlation between the frequency of the T allele in C/T SNPs and differential methylation, but it was mainly driven by hypermethylated DMRs and explained only 1 % of the variation, making it a plausible false positive (Fig. 2b). Together, this indicates that the overall difference in methylation between the cortisol groups were independent of the genetic variation in close proximity to the regions of interest. It must be emphasized that this does not mean that genetic variation in epigenetic regulatory elements elsewhere in the genome (e.g., genes that affect global DNA methylation) could have influenced our results.

Studies have also shown that blood cell types differ in DNA-methylation marks and may under some circumstances explain variability in methylation between individuals [28, 29]. To control for this, we compared the location of our *p* < 0.0001 DMRs (*n* = 852) with two independent reports listing the location of DMRs between blood cells [29, 30]. None of the cell-type-specific regions overlapped with our DMRs, and only few genes closest to the cell-type-specific DMRs overlapped with the genes closest to our DMRs (Fig. 2c). Together, this strongly indicates that the genome-wide hypomethylation seen in Hi children were not affected by cell-type methylation bias.

To further validate our experiment, three differentially methylated regions were also picked and successfully verified by bisulfite pyrosequencing: PRDM14 (rank 1), CACNA1S (rank 7), and BRCA1 (rank 89) (see “Methods” section for details).

### Differential methylation targets SINE retrotransposons

**Fig. 3:**
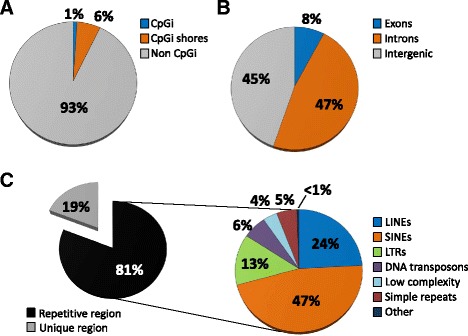
Differentially methylated regions were located in intragenic repetitive regions. **a** Circle diagrams shows the proportion of the *p* < 0.0001 DMR subset (*n* = 852) that overlapped with CpG islands (CpGi), **b** genes, and **c** repetitive elements

Broad scale age- and cancer-related hypomethylation often occur in repetitive regions outside CpG islands, which is believed to reactivate transposable elements leading to lower genome integrity, and possibly increased vulnerability to disease [22, 27]. If cortisol-associated hypomethylation arises through similar mechanisms, we would expect our DMRs to be located in these types of regions. In support of this, the *p* < 0.0001 DMR subset was generally depleted of CpG islands (Fig. 3a), was primarily found within gene regions (Fig. 3b), and contained some sort of repetitive element (Fig. 3c), where short interspersed nuclear elements (SINE; a class of retrotransposons) were particularly abundant. To assess the strength of the association between SINEs and DMRs, we performed a series of tests where the observed spatial relationships between different repeat classes and DMRs were tested against permutated genomic coordinates of the same spatial intervals. This did reveal an association between hypomethylated DMRs and SINEs, but the relationship was even stronger in hypermethylated DMRs (Table 1; Additional file 1: Figure S2A). Contrary, hypomethylated DMRs were strongly associated with genes, while hypermethylated DMRs were not (Table 1; Additional file 1: Figure S2B). There were also negative relationships between both hypo- and hypermethylated DMRs and long interspersed nuclear elements (LINEs, Table 1; Additional file 1: Figure S2C), as well as low complexity DNA (regions with very high C/G or A/T content; Table 1; Additional file 1: Figure S2D). As a control, we also analyzed CpG islands (Table 1; Additional file 1: Figure S2E), which showed neither a positive nor negative relationship to the DMRs. Together, this indicates that cortisol-associated differential methylation generally targets SINEs while avoiding other types of repeats. Furthermore, hypo- and hypermethylated regions had very different affinities for gene regions which indicate that they are discriminated by separate mechanisms.

**Table 1:** Spatial relationships between genomic intervals of top DMRs and selected genomic features

		*Hypomethylated (n = 507)*								*Hypermethylated (n = 345)*							
*Statistical concept*	*Interpretation*	*SINE*	*LINE*	*LTR*	*DNAt*	*snRNA*	*Low*	*CpGi*	*Genes*	*SINE*	*LINE*	*LTR*	*DNAt*	*snRNA*	*Low*	*CpGi*	*Genes*
Reference population	Number entities within each class	1769839	1480369	708210	456948	4285	367822	27718	23033	1769839	1480369	708210	456948	4285	367822	27718	23033
Relative KS p-value	Relative relationship between interval midpoints	<0.05	n.s.	n.s.	n.s.	n.s.	<0.1	n.s.	<0.1	<0.0001	<0.01	n.s.	n.s.	n.s.	<0.05	n.s.	n.s.
	Type of relationship	Positive	–	–	–	–	Negative	–	Positive	Positive	Negative	–	–	–	Negative	–	–
Jaccard Measure p-value	Ovelaps between intervals	< 0.0001	<0.01	<0.1	n.s.	n.s.	<0.05	n.s.	<0.0001	<0.0001	<0.001	n.s.	n.s.	n.s.	n.s.	n.s.	<0.1
	Type of overlap	Positive	Negative	Positive	–	–	Negative	–	Positive	Positive	Negative	–	–	–	–	–	Positive

Test statistics were obtained using the GenometriCorrelation package in R/Biocunductor. For more information about the different tests see Favorov *et al*. 2012. Both KS (Kolmogorov-Smirnov) and Jaccard Measure *p*-values were obtained by permuting the obeserved results 10 000 times over the human genome. *SINE* Short interspersed nuclear elements, *LINE* Long interspersed nuclear elements, *LTR* Long terminal repeats, *DNA*t DNA transposon, *snRNA* Short nuclear RNA repeats, *Low* Low complexity DNA, *CpGi* CpG island

### High cortisol is associated with extreme types of variation

Since repeat regions are commonly fully or close to fully methylated in the human genome, we would expect the DMRs to be associated with regions saturated with methylation. Our bisulfite pyrosequencing experiment supported this by generally showing highly methylated CpGs (on average 87.4 %). A region in *BRCA1* illustrates this by having 97.3 % mean methylation, where loss of methylation were only verified in Hi girls (Additional file 1: Figure S3). The loss was also accompanied by increased variation in this group, since the other groups experienced a “ceiling effect.” MeDIP-seq experiments are normally limited to relative methylation analysis, which are not suitable for saturation analysis due to a lack of an upper limit. Nevertheless, it is possible to study the ceiling effect in our MeDIP-seq. data by using the coefficient of variation (CV) as an indirect indication of saturation. CV is the unitless ratio of the standard deviation to the mean, and is commonly used to compare variance between independent variables that may differ in scale and averages. In theory, if for instance hypomethylated DMRs is a result of Hi children losing methylation at normally saturated sites, this would be reflected as a higher CV, since the CV in Lo children is affected by the ceiling effect illustrated in the *BRCA1* example. The opposite relationship would be seen in hypermethylated DMRs if they also targets SINEs that are normally saturated with methylation. In line with this hypothesis, the hypomethylated DMRs in Hi were indeed significantly associated with increased variation compared to the same DMRs in Lo children, and vice versa for hypermethylated DMRs (Additional file 1: Figure S4). Interestingly, this was mainly due to Hi children showing more variation in hypomethylated and less variation in hypermethylated DMRs compared to Lo children, which were more stable. This suggests that Hi children experience stronger fluctuations in epigenetic tones, pushing the DMRs to the extremes either by relaxation or supersaturation.

### Differential methylation in regions previously associated with aging

**Fig. 4:**
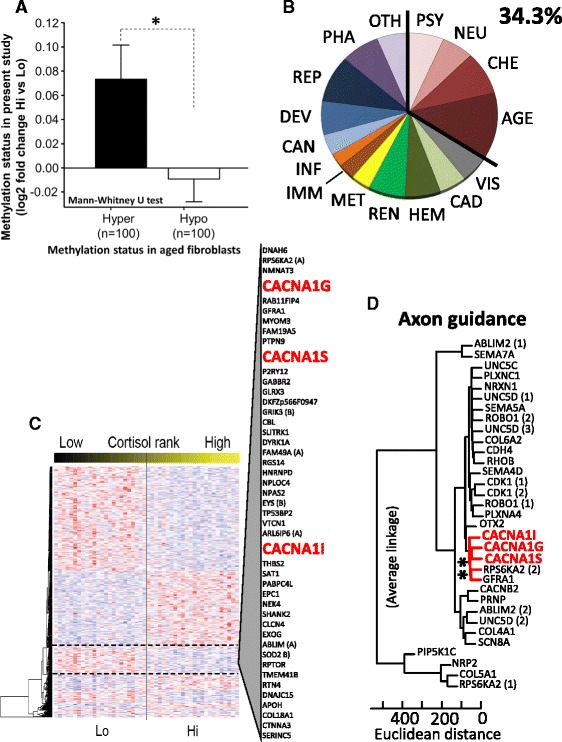
Functional analysis reveals links between DMRs and aging, neurodevelopment, and calcium transport. **a** Bar diagram shows differential methylation between Hi and Lo in the 100 strongest peaks previously reported as either hyper- or hypomethylated in aged fibroblast cells [34]. **b** Shows the normalized distribution of disease classes associated with the genes located closest to the *p* < 0.0001 DMRs (*n* = 407). Normalization was done to control for publication bias, by dividing the number of DMR genes associated with a disease class with the total number of genes present within that same class. **c** Heatmap showing the same subset of the DMRs as in **b**. Columns represent normalized counts of each child and are ordered by hair cortisol rank. Rows represents median centered Z-scores of individual DMRs, where the row order has been determined by hierarchical cluster analysis (euclidean distance; also presented as a dendogram to the right). **d** Cluster analysis of DMRs associated with genes involved in axon guidance (GO:0007411). *Red* indicates a significantly robust cluster determined by bootstrap resampling. *Hi* high hair cortisol, *Lo* low hair cortisol, *PSY* psychology, *NEU* neurological, *CHE* chemical dependence, *VIS* vision, *AGE* aging, *CAD* cardiovascular, *HEM* hematological, *REN* renal, *MET* metabolic, *IMM* immune, *INF* infection, *CAN* cancer, *DEV* development, *REP* reproduction, *PHA* pharmacogenomic, *OTH* other

There have been a number of human genome-wide studies that report differentially methylated regions associated with aging [31–34]. Since stress has been hypothesized to increase the rate of biological senesces [4], it would be interesting to see if the DMRs between Hi and Lo overlap with genomic regions associated with methylation changes in senescens. Unfortunately, most of these studies are not comparable to our data since microarray technologies have been used that are biased towards CpG islands and poor in retrotransposon coverage. To our knowledge, only one study has used a similar method to the MeDIP-seq used in our study. Hänzelmann et al. used MethylCap-seq to study the effect of senescence in fibroblast cell cultures [34]. Similar to the difference between Hi and Lo children, derived fibroblast showed more hypomethylated regions compared to young cells. Considering the top DMRs in both studies, only 10 overlaps were found (<2 %) between our *p* < 0.0001 DMR subset (*n* = 852) and the 16,962 unique DMRs identified in the fibroblast study. The lack of overlap was not a surprise since even between biological replicates within the fibroblast study, there was a very limited overlap [34]. Nevertheless, when we instead investigated the 100 strongest hypo- and hypermethylated regions in the fibroblast study (as identified by highest peak) in our MeDIP-seq data, Hi children showed significantly more methylation in fibroblast hypermethylated than in hypomethylated regions (Fig. 4a). This suggests that, even though the grand effect of high cortisol levels is not present in senesces-related regions identified in the fibroblast cell cultures, there is a statistical similarity between the datasets that makes it important to further study the epigenetic link between stress and aging.

### Association with genes involved in calcium transport and neurodevelopment

To identify more mechanistic clues, we annotated the top 852 DMRs to their closest gene. Fifty-one percent of the DMR associated genes had previously been linked to disease (*n* = 407; Fig. 4b). Even though several disease classes were represented here, we noted that genes associated with neuropsychiatric disorders, especially those related to aging, were in majority (see Additional file 1: Figure S5). This link was confirmed when performing more in-depth gene enrichment analysis, where for instance genes with functional terms like “neurodevelopment” and “axon guidance” were highly enriched within the subset (Additional file 1: Figure S6). The gene functional analysis also revealed a significant enrichment of genes that previously have been shown to interact with decitabine and azacitidine (drugs known to cause DNA hypomethylation), as well as genes involved in calcium transport (Additional file 1: Table S4). Notably, 25 % of all voltage-gated calcium channel subunits were represented among the *p* < 0.0001 DMR associated genes.

It is well-known that excessive exposure to glucocorticoids may affect renal excretion and intestinal absorption of calcium, which together with changes in osteoblastic function is regarded as the main pathogenesis of glucocorticoid-induced osteoporosis [35]. This is often seen in asthma patients given chronic high doses of glucocorticoids [36] or patients with endogenous Cushing’s syndrome, caused by an endogenous overproduction of glucocorticoids [37]. A dysfunctional calcium transport, specifically related to voltage-gated calcium channels, has also been linked to age-related cognitive disorders [38], cardiovascular diseases [39], and diabetes [40]. Thus, hypothetically, changes in epigenetic regulation of calcium channels may at least partly explain why early life stress affects vulnerability to these diseases in adulthood.

### Consensus sequence in voltage-gated calcium channel genes

Since there was an overrepresentation of hypomethylated DMRs in the Hi children and since large scale demethylation events, in addition to a dysfunctional calcium transport, has been associated with stress-related diseases (see above), we were particularly interested in hypomethylated calcium channels. We noted that three of the DMRs associated with voltage-gated calcium channel genes (*CACNA1S*, *CACNA1I*, *CACNA1G*) had similar methylation patterns across all children, both when considering the disease-associated DMRs (Fig. 4c) and even clearer when only considering genes involved in axon guidance (Fig. 4d). This suggests that these genes are epigenetically controlled by the same mechanism, involving the same transcription factors.

**Fig. 5:**
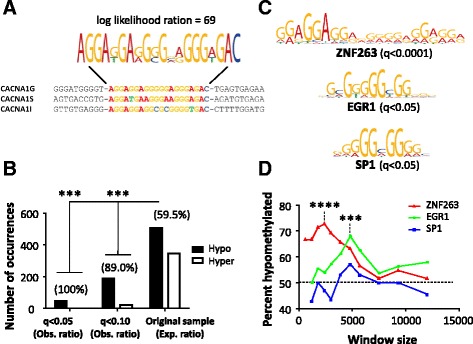
ZNF263 binding is overrepresented in hypomethylated regions both *in silico* and *in vitro*. **a** Shows the most similar sequences within the co-methylated calcium channel DMRs. **b** *Bars* show the number of hypo- and hypermethylated DMRs among the *p* < 0.0001 subset (*n* = 852) having significantly similar sequences to the calcium channel consensus sequence presented in **a**. Q-value thresholds represent false discovery rate-adjusted *p* values attained from log-odds scores of the sequence similarities. A chi-square test was used to compare the observed distributions at *q* < 0.05 and *q* < 0.1 to the expected distribution, as shown by the percent hypomethylation of the original DMRs. **c** The calcium channel consensus sequence was significantly similar to three predicted zinc-finger transcription factor binding site sequences (motifs). **d** Shows the percent representation of hypomethylated DMRs that overlapped with transcription factor binding sites as measured by chromatin immunoprecipitation sequencing and posted within the Encode project. To allow for a progressive increase in genomic window size, the top 300 hypomethylated DMRs were compared to the top 300 hypermethylated DMRs, where the *dotted line* represents the expected overlaps under random conditions. Note that the ZNF263 binding occurs close to the hypomethylated DMRs, while the EGR1 binding is more distantly located, becoming significantly associated only at a 5 kb distance. ****p < 0.0001 and ***p < 0.001 represents chi-square tests. The consensus/motif analyses were performed using the MEME suite tools; **a** = MEME, **b** = TOMTOM, and **c** FIMO (http://meme-suite.org/)

To investigate if the regions associated with the co-methylated calcium channels had a common transcription factor binding site, we used the MEME suite of bioinformatics tools [41–43] to search for consensus sequences (sequences with high resemblance) in or in the immediate vicinity (±300 bp) of the associated DMRs. Figure 5a shows the strongest consensus sequence present in all three calcium channel DMRs. When further searching for presence of a similar sequence in the *p* < 0.0001 DMR subset, it was clear that this consensus sequence was not only present in the calcium channel DMRs, but generally overrepresented in hypomethylated regions of Hi children (Fig. 5b). In fact, 39 % of the hypomethylated DMRs contained a similar sequence, while only 7 % of the hypermethylated DMRs did so. This suggests that the sequence is generally targeted by cortisol-associated hypomethylation in these children.

### ZNF263 binding sites is targeted by hypomethylation

To get an indication of what transcription factor may bind the calcium channel consensus sequence, we compared this sequence to predicted transcription factor binding sequences (motifs). At least three zinc-finger transcription factors were found to have significantly similar binding sequences: ZNF263, EGR1, and SP1 (Fig. 5c).

To further address the question which of the transcription factors were more likely to be affected by hypomethylation in these regions, we compared our DMRs to known *in vitro* ZNF263, EGR1, and SP1 binding sites acquired from previous chromatin immunoprecipitation experiments in cell cultures posted within the Encode project. We found that ZNF263 binding was associated with our hypomethylated DMRs on a proximate genomic level, while EGR1 binding occurred more distantly to the identified regions (Fig. 5d). Since DNA methylation is tightly linked to the local chromatin configuration which in turn determines the accessibility of the DNA strand, this may indicate a coordinated regulatory relationship between these transcription factors, but clearly, further investigation is needed here.

Since most of our DMRs contained transposable elements, we would expect ZNF263 to bind similar repeat regions, if there is an association. Therefore, we analyzed the occurrence of repeat/transposable elements within the ZNF263, as well as EGR1 and SP1, binding regions previously identified in Encode chromatin immunoprecipitation experiments. ZNF263 binding was indeed more associated with repetitive regions than EGR1 and SP1 binding (Additional file 1: Figure S7). As in our DMRs, SINEs were the most abundant repeat family in ZNF263 binding regions.

### General discussion

To our knowledge, this is the first prospective study on the genome-wide epigenetic impact of stress in preschool children. This age is characterized by fast intellectual, social, and emotional development that usually influences the individual for life. By using hair cortisol as an indicator of chronic stress in otherwise healthy children, we show that high cortisol is associated with a decrease in DNA methylation at ZNF263 binding sites and targets SINE retrotransposons across the human genome.

Little is known about the function of ZNF263, except that it is predicted to have a repressive effect on gene transcription and often binds intragenic regions [44]: a feature that it shares with the DMRs between the Hi and Lo children. We also show that its binding sites generally overlap with repetitive elements, particularly with SINEs, which is also a feature that it shares with the DMRs. Fast evolving, species specific zinc-finger proteins that contain KRAB domains (KZNF) have recently been shown to coevolve with novel retrotransposons, in what seems to be an “arms race,” indicating an important role for KZNFs as repressors of retrotransposon activity [45]. In contrast to the KZNFs just mentioned, ZNF263 is conserved among mammalian species, which indicates that it has lost its role in acute host defense, but instead may have acquired other important functions.

SINEs have previously been associated with highly expressed genes [46]. Most of the DMRs between Hi and Lo children, particularly those associated with disease genes, were completely depleted of DNA methylation in their first exon (Additional file 1: Figure S8): a trait that predicts high gene activity even better than promoter methylation [47]. If ZNF263 has maintained its repressive role as a KZNF, hypomethylation at its binding sites would predict a repressive effect on the regulation of SINE-linked genes that are normally highly expressed (such as many housekeeping genes). What role this may play in disease is too early to tell, but it is critical to further study how DNA methylation affects KZNF binding and SINE linked gene expression.

The repressive effect of KZNFs is dependent on the recruitment of KRAB-associated protein 1 (KAP1). In turn, KAP1 recruits the histone methyltransferase SETDB1, heterochromatin protein 1(HP1), and the NuRD histone deacetylase complex, which epigenetically inactivates the KZNF binding region [44, 45]. Thus, it is possible that the loss of DNA methylation at ZNF263 binding sites seen in stressed children is directly mediated by a loss of ZNF263 itself. If this is the case, it is tempting to speculate that therapies that increase the level of ZNF263 may regain DNA methylation in retrotransposons, leading to increased genome integrity and a positive effect on patients experiencing age and stress-related diseases, as well as cancer patients. Such therapies may potentially even affect the aging process: a brave hypothesis requiring extensive further research.

Our results were inconclusive in what role methylation at EGR1 binding sites plays in the stress of these 5-year-old children. This immediate early gene is well-known for its evolutionary conserved activation by different kinds of environmental stimuli, including acute stress [19, 20]. We did not detect any effect of high cortisol on the methylation of EGR1 binding sites in the *NR3C1* gene, which encodes the glucocorticoid receptor that uses cortisol as a ligand. According to the Encode chromatin immunoprecipitation data, ZNF263 binding does not occur anywhere near the *NR3C1* gene. This indicates that what we observe prospectively in the blood of high hair cortisol, but otherwise healthy children, is an independent stress-related mechanism relative to what others have observed retrospectively in for example brains of adult males [18] or in blood of adolescents [48] with a history of childhood abuse.

We have already addressed some of the limitations of this type of human study (see “Differential methylation not explained by genetic heterogeneity or stress induced bias in cell types” section). In addition, two more limitations, interrelated and specific for this study must be discussed: the low sample size and the sequencing depth. A sample size of 48 children that are mixed in gender is not ideal, particularly since gender is a significant factor in early life adversity (for recent relevant reviews, see [49–51]). That we only detect 10 significant differentially methylated regions (after false discovery rate correction) might be an effect of mixed gender groups where all sex-dependent DMRs have been statistically blocked by a cofactor in our model. Analyzing the data from a gender perspective was not an option because it would create even smaller study groups that would decrease statistical power to unacceptable proportions. On the other hand, that we find our main effects in repeat regions, genomic features that are found in many copies across the genome and often are highly methylated, is encouraging in this type of study. With such genomic features, evidence is gathered at many loci, which make the findings not heavily dependent on specific loci in individual samples. Thus, a low sample size may reliably detect the effects in this type of region. Highly methylated regions are also preferably studied with immunoprecipitation-based methylome sequencing, such as MeDIP-seq or MethylCap-seq, since very low methylated regions, such as some CpG islands, need very high sequencing depths to build up the amount of sequence reads needed to accurately detect differences between study groups. Therefore, it is possible that changes at low methylated CpG islands have “passed under our radar.”

In addition to the epigenetic limitations, it must also be emphasized that studying hair cortisol in young children is relatively novel. Even though clear correlations between experienced stress and hair cortisol have been validated extensively in adults, only a few studies have validated the method in young children [12, 52].

## Conclusions

Our data shed light over the initial epigenetic changes associated with stress during childhood and may help to understand how early adverse experiences epigenetically predispose an individual for certain diseases. We propose that this mainly involves demethylation processes, similar to those seen in aging, which targets SINE retrotransposons as well as specific zinc-finger protein binding sites. We have identified one of these proteins, ZNF263, and appeal to future investigators to study this transcription factor in the development of the many complex diseases associated with stress and aging.

## Availability of supporting data

The sequencing data supporting the results of this article is available in the European Genome-phenome Archive (EGA; hosted by the EBI) repository under accession number EGAS00001001099 (www.ebi.ac.uk/ega/studies/EGAS00001001099).

## Methods

### Ethical statement and Data access

The Research Ethics Committee at The Faculty of Health Sciences, Linkoping University, Sweden, has approved the ABIS study at several occasions initially Dnr 1996/287 to the latest Dnr 2013/253-32. Written consent to participate in the study was given by the principal persons with parental responsibility. Sequence data has been deposited at the European Genome-phenome Archive (EGA; hosted by the EBI) under accession number EGAS00001001099 (www.ebi.ac.uk/ega/studies/EGAS00001001099). To protect the personal integrity of the subjects according to Swedish law, data access is controlled by a committee.

### Subjects and Study design

Sample collection has been described elsewhere [12]. In short, hair and blood samples were collected from a subsample of children participating in the All Babies in South-East Sweden (ABIS) project, a cohort initially including 17,000 children born between 1997 and 1999, followed prospectively with questionnaires and biological samples at regular intervals to identify environmental risk factors for complex diseases. In Additional file 1: Table S1, sociodemographic data on the subsample of ABIS children enrolled in the present study are presented.

### Hair cortisol measurement

Cortisol concentrations were measured using a competitive radioimmunoassay in methanol extracts of pulverized hair. A 3-mm thick and 3-cm long hair sample, weighing 5–6 mg, was cut close to the scalp from the posterior vertex area of the head. Samples were finely cut into tubes containing 0.5 mm stainless steel beads. To produce a fine hair powder, samples were put into aluminum cylinders, frozen in liquid nitrogen for 2 min, and minced with a Tissue Lyser II (Retch) at 23 Hz for 2 min. Cortisol was extracted by adding 1 ml of methanol, followed by incubation on a horizontal shaker at room temperature for 10 h, where the steel beads were kept in constant motion. Tubes were centrifuged for 1 min at 13,000 rpm and 4 °C. Of the supernatant, 800 μl was then moved to another tube and lyophilized in a SpeedVac Plus SC210A (Savant) for 2 h. Samples were then dissolved in radioimmunoassay buffer and analyzed as described elsewhere [53]. The intra-assay coefficient of variation for the radioimmunoassay was 7 % at 10 nmol/l. The antiserum cross reacted 137 % with 5α-dihydroxycortisol, 35.9 % with 21-deoxycortisol, 35.9 % with prednisolone, but less than 1 % with endogenous steroids.

### Library preparation and MeDIP

We used a similar method as described in detail by Taiwo et al. [54]. If not otherwise stated, kit manufacturer recommendations were always followed. DNA from 48 whole blood samples (29 females and 19 males) was extracted using the FlexiGene DNA kit with RNase treatment (Qiagen). From each sample, 1.9 μg DNA was diluted in 85 μl buffer FG3 (Qiagen) and fragmented using a Bioruptor Standard sonicator (Diagenode). A mean fragment size of 225–250 bp was verified on a Bioanalyzer (Agilent). Fragmented samples were DNA end-repaired, dA-tailed, and adapter ligated using the NEBNext® DNA Library Prep Master Mix Set for Illumina® kit (New England Biolabs) with AMPure XP bead cleanups (Beckman Coulter). MeDIP was then performed overnight in five different batches (balanced based on group) using the MagMeDIP kit (Diagenode). Input control samples without antibody were saved overnight at 4 °C. Immunoprecipitated methylated fragments, as well as input samples, were recovered using the IPure kit (Diagenode). Using the NEBNext® Multiplex Oligos for Illumina® (Index Primers 1–12) kit (New England Biolabs), sequence barcoding was introduced by high fidelity amplification. The products were run on a 2 % agarose gel electrophoresis, fragments of 300–350 bp sizes were cut out of the gel using clean scalpels, and purified using MiniElute Gel DNA extraction kit (Qiagen). Fragment purity and sizes were verified on a Bioanalyzer (Agilent). All samples were diluted to 10 nM in RNase free water, verified by Qubit 2.0 flourometer (Invitrogen). Sequencing was performed on a HiSeq 2500 (Illumina), using 100 bp paired-end reads, resulting in a mean depth of 36.87 M reads (max = 94.30 M, min = 20.97 M).

### Analysis of DMRs

Information about the main bioinformatics tools used is summarized in Additional file 1: Table S2. The hair cortisol measurements were used to rank each subject into a high (Hi > 8.9 fmol/mg) and a low (Lo < 8.9 fmol/mg) group (total *n*_High_ = 24, *n*_Low_ = 24, girls *n*_High_ = 16, *n*_Low_ = 13, boys *n*_High_ = 8, *n*_Low_ = 11). FastQ quality thresholds were set to the Illumina 1.8+ standard. Demultiplexing and conversion were done using CASAVA v1.8.2 (Illumina). The Burrows-Wheeler Alignment tool and Picard [55] were used to map the reads to the hg19 human reference genome and to remove PCR duplicates using default settings. Analysis was done using different packages in the R/Bioconductor software environment (http://www.bioconductor.org/). MeDIP quality was assessed within the MEDIPS package [56]. All 48 samples were saturated and showed MeDIP typical CpG coverage. CpG Enrichment scores (relH) for MeDIP samples were >2.0 (mean = 2.53, max = 3.21, min = 2.05), while input control samples (*n* = 4) were closer to 1.0 (mean = 1.34, max = 1.46, min = 1.25). Whole-genome methylation analysis was done by tiling 300 bp windows across the human genome using the GenomicRanges package [57]. A filter was applied to maintain ranges that had more than five counts in at least half of the samples, and each count table was then normalized using the full quantile normalization procedure in EDASeq [58]. Differential methylation analysis between Hi versus Lo was done on filtered normalized count data within edgeR [59], where a generalized linear model was applied to block out any remaining effect of gender and immunoprecipitation efficiency (IP-batch), an effect commonly seen in immunoprecipitation experiments [60]. *p* values were generated using the tagwise dispersions within edgeR and were false discovery rate (FDR)-corrected using the Benjamini and Hochberg (BH) method. Each DMR was annotated to the closest gene in relation to transcription start sites.

### Post DMR data analysis

CV analysis was done by first generating the CVs of the normalized read counts of individual DMRs within each group (Hi/Lo) and then comparing hyper- and hypomethylated CVs with a Mann-Whitney U test. For gene functional analysis of the DMRs, we used WEB-based GEne SeT AnaLysis Toolkit (WebGestalt) [61]. For more specific analysis, genomic feature data (introns, exons, CpG islands) was downloaded from UCSC table browser, gene/disease class annotations were downloaded from Disease Association Database [62], while repetitive regions were collected from RepeatMasker (http://www.repeatmasker.org/). Cluster analysis with dendograms and heatmaps was done using hierarchal clustering with euclidean distances generated by the gplots [63] and pvclust [64] R packages, where individual cluster robustness was determined by approximately unbiased *p* values calculated by multiscale bootstrapping (10,000 permutations) within pvclust. We used the GenometriCorr R package [65] to evaluate whether the position of our DMR intervals where statistically associated with different repeat classes in genomic space. Provided with the genomic intervals of individual DMRs and repeats, we calculated the overlap and relative genomic distances (midpoint to midpoint) between the DMRs and the repeats. By permuting the intervals across the human genome 10,000 times, a null distribution was created and compared to the observed distribution through a Kolmogorov-Smirnov test for relative distance relationships, and Jaccard’s index for overlap relationships. Motif analysis on clustered calcium channels (CACNA1S/G/I) was done using MEME [41] where log likelihood ratios was computed given the motif model versus its probability given the 0-order Markov background model of the provided nucleotide frequency of each DMR (±300 bp). The consensus motif with highest score was then exported to TOMTOM [42] for similarity analysis of known transcription factor binding motifs within the Jaspar Core 2014 Vertebrates and UniProbe Mouse (UniProbe Mouse results not shown) databases. FIMO [66] was used for enrichment analysis of the occurrence of similar motifs in the 900 bp regions centered on each DMR of the *p* < 0.0001 list. Only false discovery rate-adjusted *q* values were considered in both the TOMTOM and FIMO analysis. For Encode TF-binding analysis, we downloaded and merged all transcription factor binding peaks for ZNF263, SP1, and EGR1 in all reported cell lines (ZNF263 = K562, HEK293-T-REx; SP1 = GM12878, H1-hESC, K562, HEPG2; EGR1 = GM12878, H1-hESC, K562) available in UCSC’s Transcription factor ChIP-seq Uniform Peaks Track (Uniform TFBS) [67].

### Validation using bisulfite pyrosequencing

We choose to verify three top-ranked DMRs with bisulfite pyrosequencing: *PRDM14* (rank 1), *CACNA1S* (rank 7), and *BRCA1* (rank 89). According to manufacturer’s recommendations, 400 ng of DNA was bisulfite-converted using the EpiTect Fast DNA Bisulfite Kit (Qiagen) and amplified with the PyroMark PCR Kit (Qiagen) using the primers in Additional file 1: Table S5. The biotinylated PCR product was then quantitatively sequenced on a PyroMark Q96 MD (Qiagen). As expected, all three DMRs correlated significantly either negatively with hair cortisol or positively with MeDIP-seq data (PRDM14_CORT_: *r* = −0.47, *p* = 0.02; CANA1S_SEQ_: *r* = 0.40, *p* = 0.006; BRCA1_CORT_ = −0.36, *p* = 0.05). It must be emphasized that this is a good result considering that the estimated target windows for our MeDIP-seq analysis is >800 bp (300 bp analysis windows overlapping 225–250 bp fragments), while bisulfite pyrosequencing targets single nucleotides.

### Genetic association in DMRs

By analyzing the mapped reads from the MeDIP-seq in the Genome Analysis Toolkit (GATK) [68], we detected 3304 suggestive single nucleotide variations located within 900 bp windows centered over the *p* < 0.0001 DMRs (*n* = 852). Genetic association filtering and analysis was performed using the default pipeline for single variant analysis in the Efficient and Parallelizable Association Container Toolbox (EPACTS; http://genome.sph.umich.edu/wiki/EPACTS) generating 2228 high quality SNPs.

## Additional file

Additional file 1:
**Supplemental file.** Supplemental information containing the following features. **Figure S1.** Individual methylation profiles over the PRDM14 gene. **Figure S2.** Overlaps between DMRs and selected genomic features. **Figure S3.** Bisulfite pyrosequencing results for the BRCA1 gene. **Figure S4.** Variation analysis (using CV). **Figure S5.** Raw frequencies of disease class representation and evidence for neuropsychiatric genes within the AGE class. **Figure S6.** Gene ontology tree. **Figure S7.** Candidate Zinc-finger binding to repetitive sequences. **Figure S8.** First exon versus last exon methylation. **Table S1.** Some demographic and social data of the participating children. **Table S2.** Summary of the main bioinformatics tools. **Table S3.** Differentially methylated regions in the whole-genome analysis. **Table S4.** A selection of the top results from the functional annotation analysis. **Table S5.** Primers used for bisulfite pyrosequencing.

## Abbreviations

BRCA1: breast cancer 1, early onset
CACNA1G: calcium channel, voltage-dependent, T type, alpha 1G subunit
CACNA1I: calcium channel, voltage-dependent, T type, alpha 1I subunit
CACNA1S: calcium channel, voltage-dependent, L type, alpha 1S subunit
EGR1: early growth response 1
Hi: children of the present study showing high levels of hair cortisol (>8.9 fmol/mg)
KZNF: KRAB domain zinc-finger proteins
LINE: long interspersed nuclear element
Lo: children of the present study showing low levels of hair cortisol (<8.9 fmol/mg)
LTR: long terminal repeats
MeDIP: methylated DNA immunoprecipitation
NR3C1: nuclear receptor subfamily 3, group C, member 1 (glucocorticoid receptor)
PRDM14: PR domain containing 14
SINE: short interspersed nuclear element
ZNF263: zinc-finger protein 263
