# Supplementary material for: High cortisol in 5-year-old children causes loss of DNA methylation in SINE retrotransposons: a possible role for ZNF263 in stress-related diseases
Source: Clin Epigenetics. 2015 Sep 4;7(1):91. doi: 10.1186/s13148-015-0123-z (PMC4559301; doi:10.1186/s13148-015-0123-z)
Supplement: Additional file 1: — Supplemental file. Supplemental information containing the following features. Figure S1. Individual methylation profiles over the PRDM14 gene. Figure S2. Overlaps between DMRs and selected genomic features. Figure S3. Bisulfite pyrosequencing results for the BRCA1 gene. Figure S4. Variation analysis (using CV). Figure S5. Raw frequencies of disease class representation and evidence for neuropsychiatric genes within the AGE class. Figure S6. Gene ontology tree. Figure S7. Candidate Zinc-finger binding to repetitive sequences. Figure S8. First exon versus last exon methylation. Table S1. Some demographic and social data of the participating children. Table S2. Summary of the main bioinformatics tools. Table S3. Differentially methylated regions in the whole-genome analysis. Table S4. A selection of the top results from the functional annotation analysis. Table S5. Primers used for bisulfite pyrosequencing. [file 13148_2015_123_MOESM1_ESM.pdf]

Index Supplemental Files

1. Supplemental figures

Fig. S1 - Individual methylation profiles over the PRDM14 gene.

Fig. S2 - Overlaps between DMRs and selected genomic features.

Fig. S3 - Bisulfite pyrosequencing results for the BRCA1 gene.

Fig. S4 - Variation analysis (using CV).

Fig. S5 - Raw frequencies of disease class representation and evidence for neuropsychiatric genes within the AGE class.

Fig. S6 - Gene ontology tree.

Fig. S7 - Candidate Zinc finger binding to repetitive sequences.

Fig. S8 - First exon versus last exon methylation.

2. Supplemental tables

Table. S1 - Some demographic and social data of the participating children.

Table. S2 - Summary of the main bioinformatics tools

Table. S3 - Differentially methylated regions in the whole genome analysis.

Table. S4 - A selection of the top results from the functional annotation analysis.

Table. S5 - Primers used for bisulfite pyrosequencing.

1. Supplemental figures

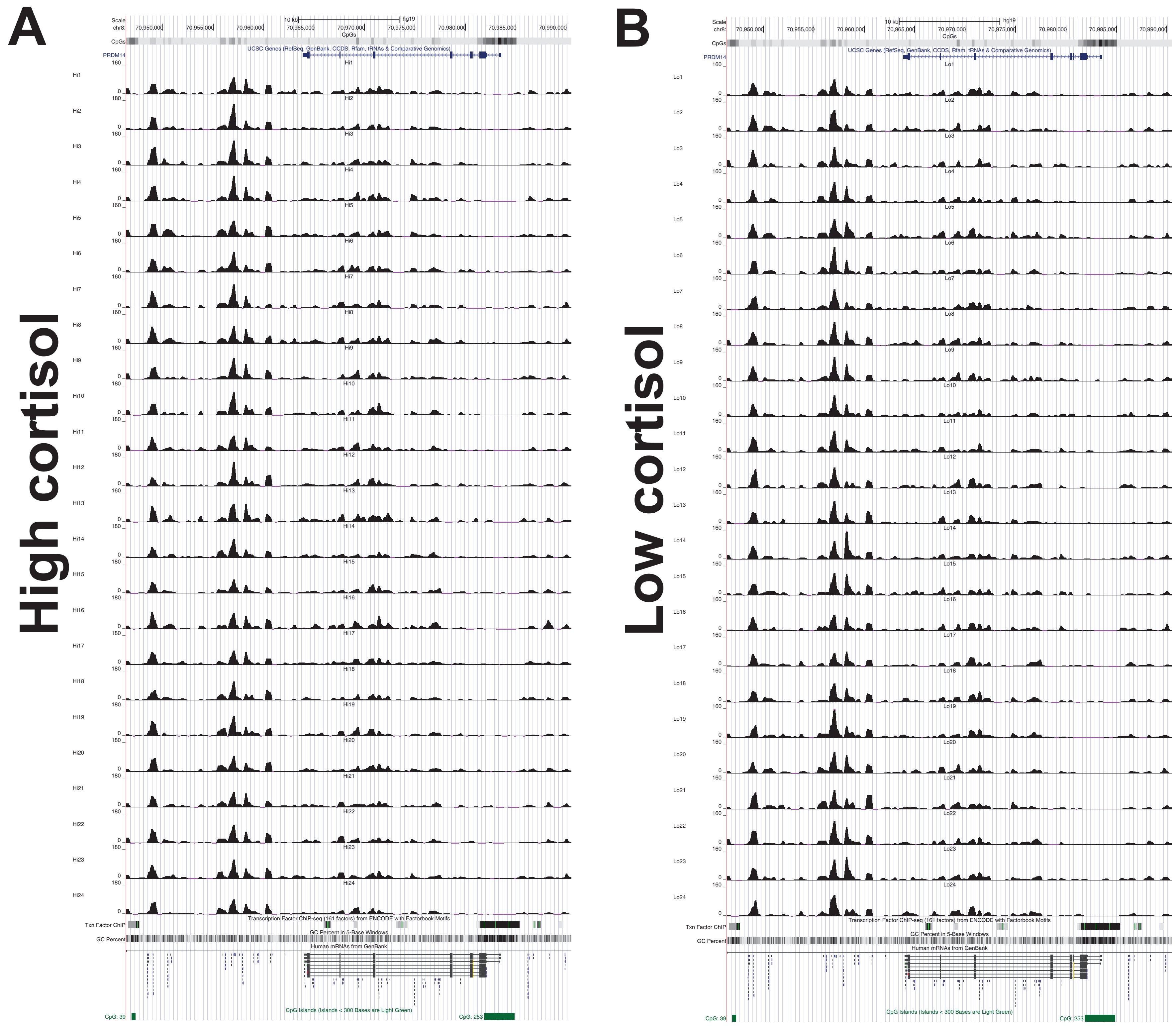

Fig. S1. Individual methylation profiles over the *PRDM14* gene. DNA methylation was measured in 300 bp windows. Top track shows relative CpG content in each window. For every methylation profile the Y-axis shows number of read counts, while the X-axis shows the position on chromosome 8 in bp. Graphs were generated and downloaded from UCSC genome browser custom tracks.

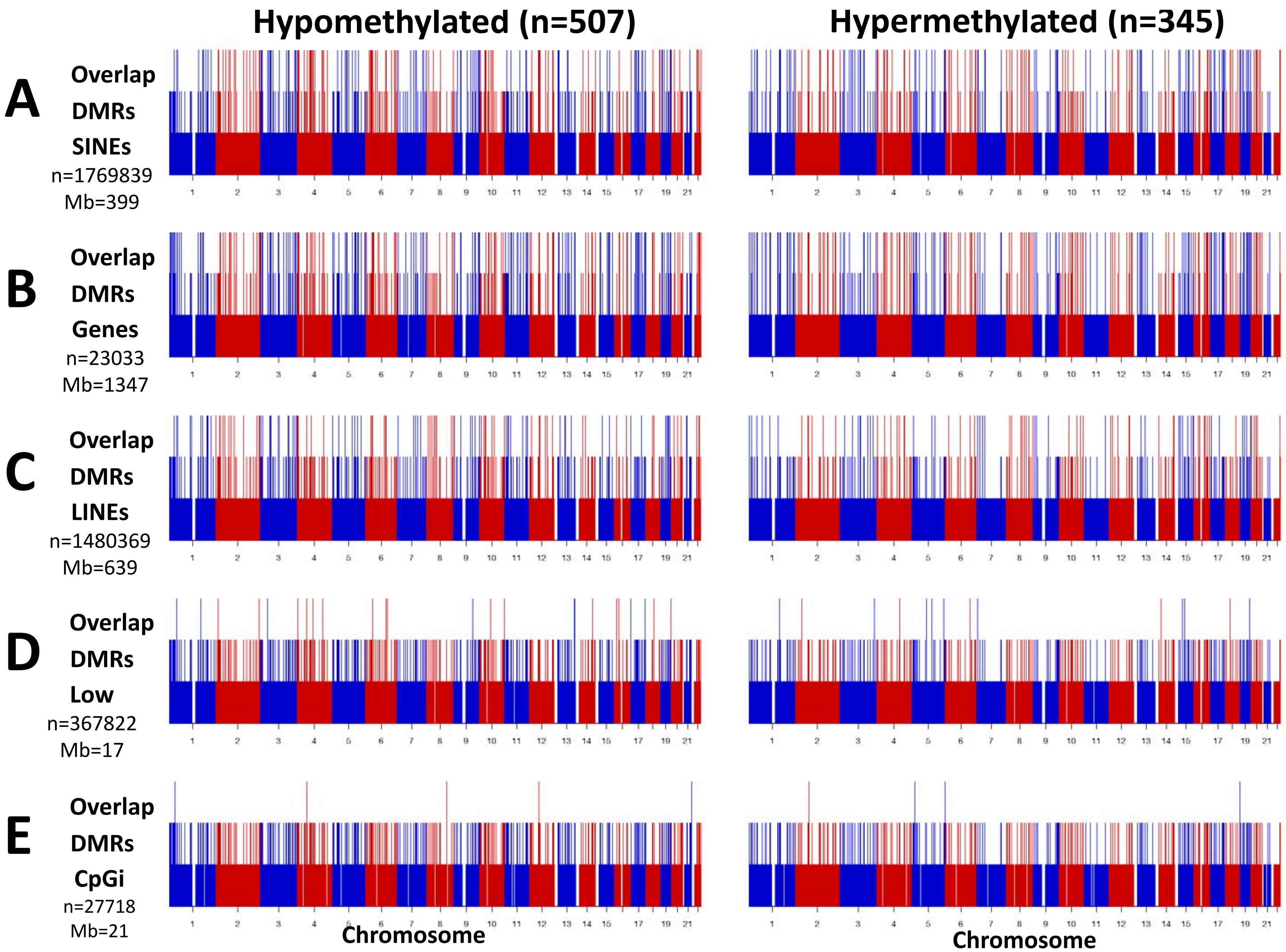

Fig S2. Overlaps between DMRs and selected genomic features. Colors divides chromosomes. SINE/LINE = Short/Long Interspersed Nuclear Element. Low = Low complexity DNA. CpGi = CpG islands. Mb = Coverage in megabases.

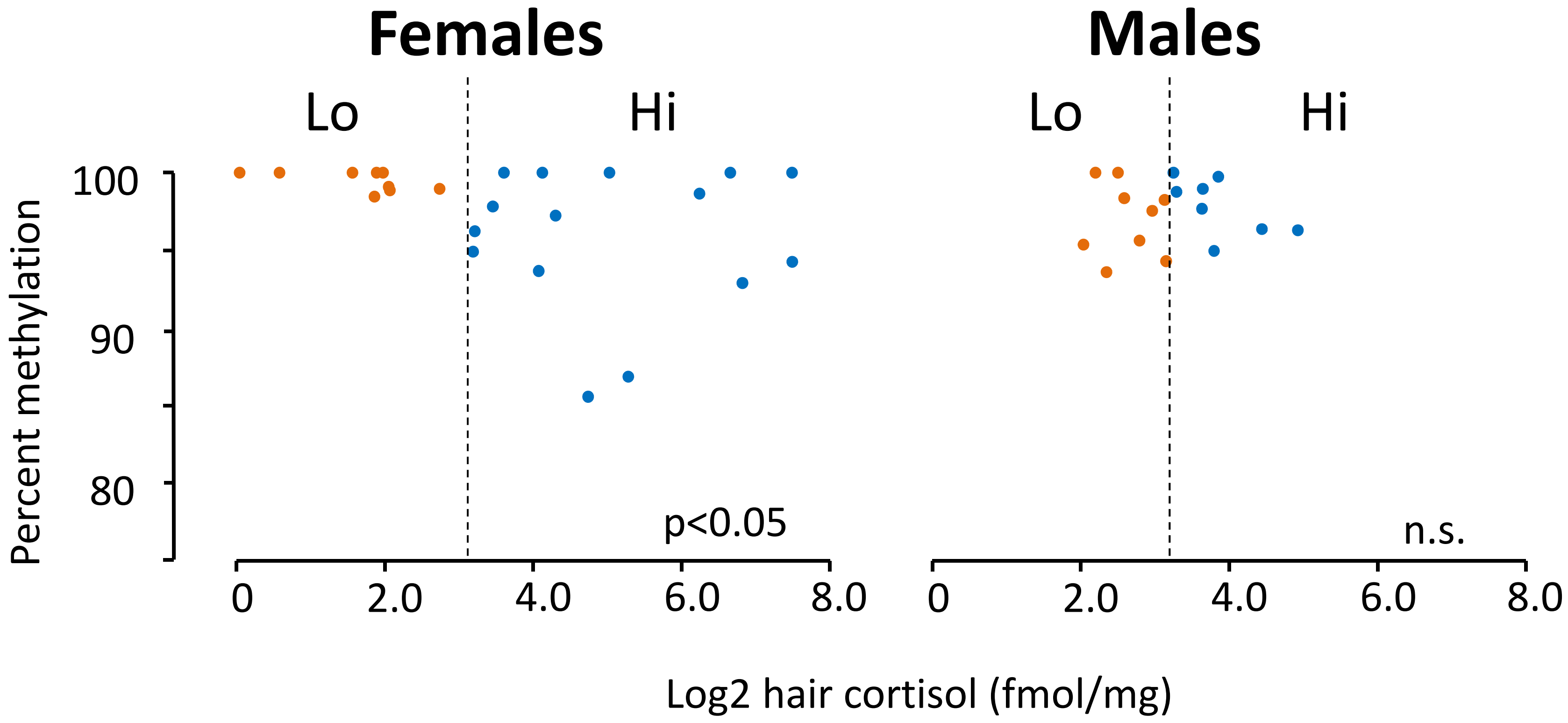

Fig S3. Bisulfite pyrosequencing results for the *BRCA1* gene. Blue represents high and red low hair cortisol children. P-value indicates results of a Mann-Whitney U test.

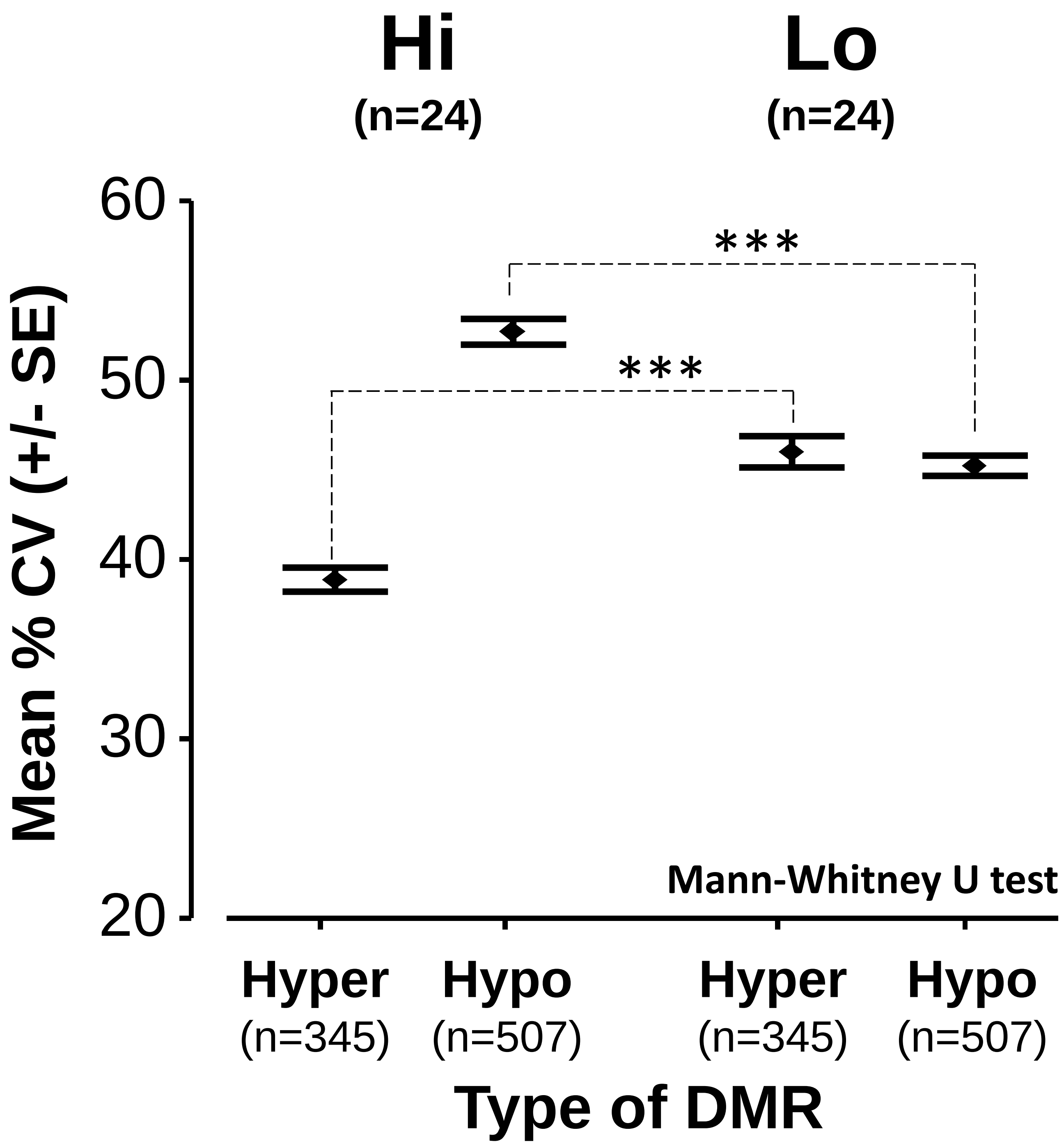

Fig S4. Comparison of the variation in DMR methylation between Hi and Lo, as measured by the Coefficient of Variation (CV). Hypo-methylated DMRs show more variation, while hypermethylated DMRs show less variation in Hi compared to Lo, where Lo is more stable across all DMRs. CVs were calculated by using the read counts of individual DMRs within each group (Hi/Lo), while standard errors (SE) were derived from the means of the hypo- and hypermethylated CVs respectively.

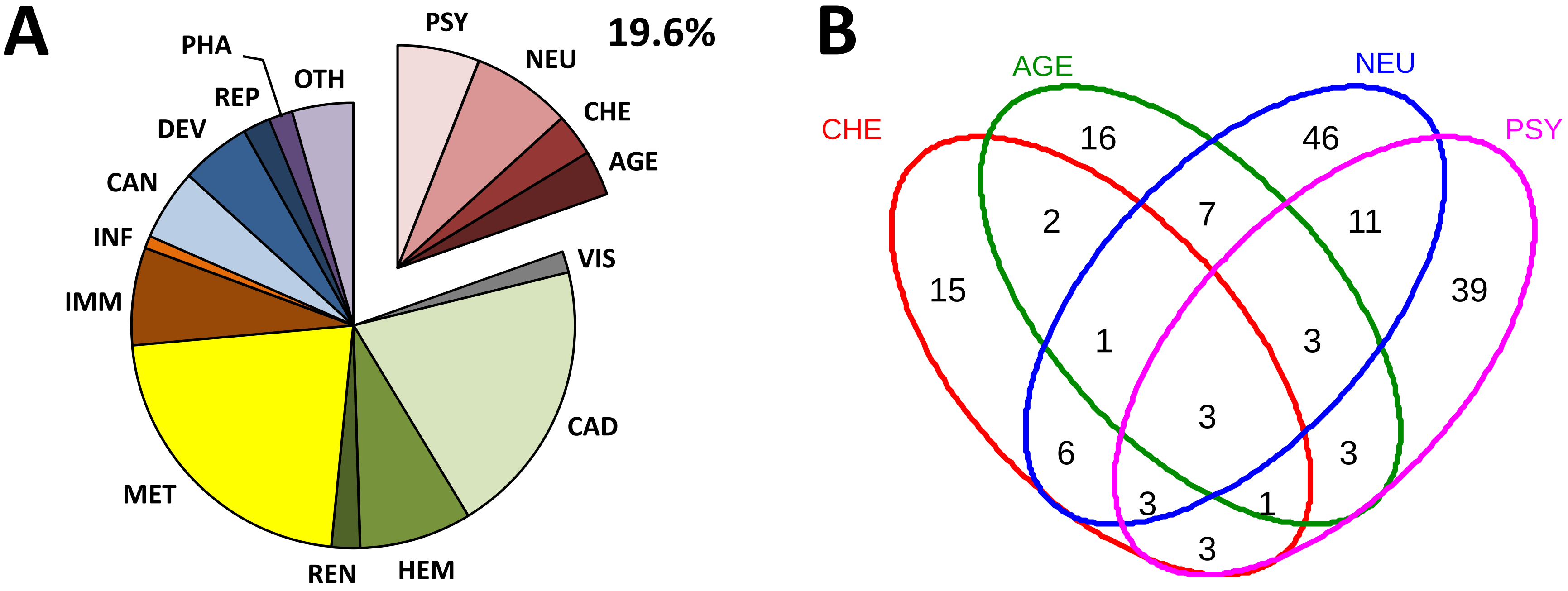

Fig S5. Raw frequencies of disease class representation and evidence for neuropsychiatric genes within the AGE class. (A) Circle diagram shows non-normalized proportions of disease classes within the p<0.0001 DMR associated genes. Note that MET and CAD are both heavily affected by publication bias containing more than 15 000 and 10 000 gene associations respectively, compared to REP and PHA that contains less than 600. Thus, normalizing with total number of listed genes within each class is appropriate (Fig. 4B in main article). (B) Wenn diagram showing gene overlaps between four disease classes. Note that the majority of PSY and NEU disease genes do not overlap, while most genes associated with AGE diseases overlap with PSY, NEU and CHE associated diseases/disorders. PSY=Psychology, NEU=Neurological, CHE=Chemical dependence, VIS=Vision, AGE=Aging, CAD=Cardiovascular, HEM=Hematological, REN=Renal, MET=Metabolic, IMM=Immune, INF=Infection, CAN=Cancer, DEV=Development, REP=Reproduction, PHA=Pharmacogenomic, OTH=Other.

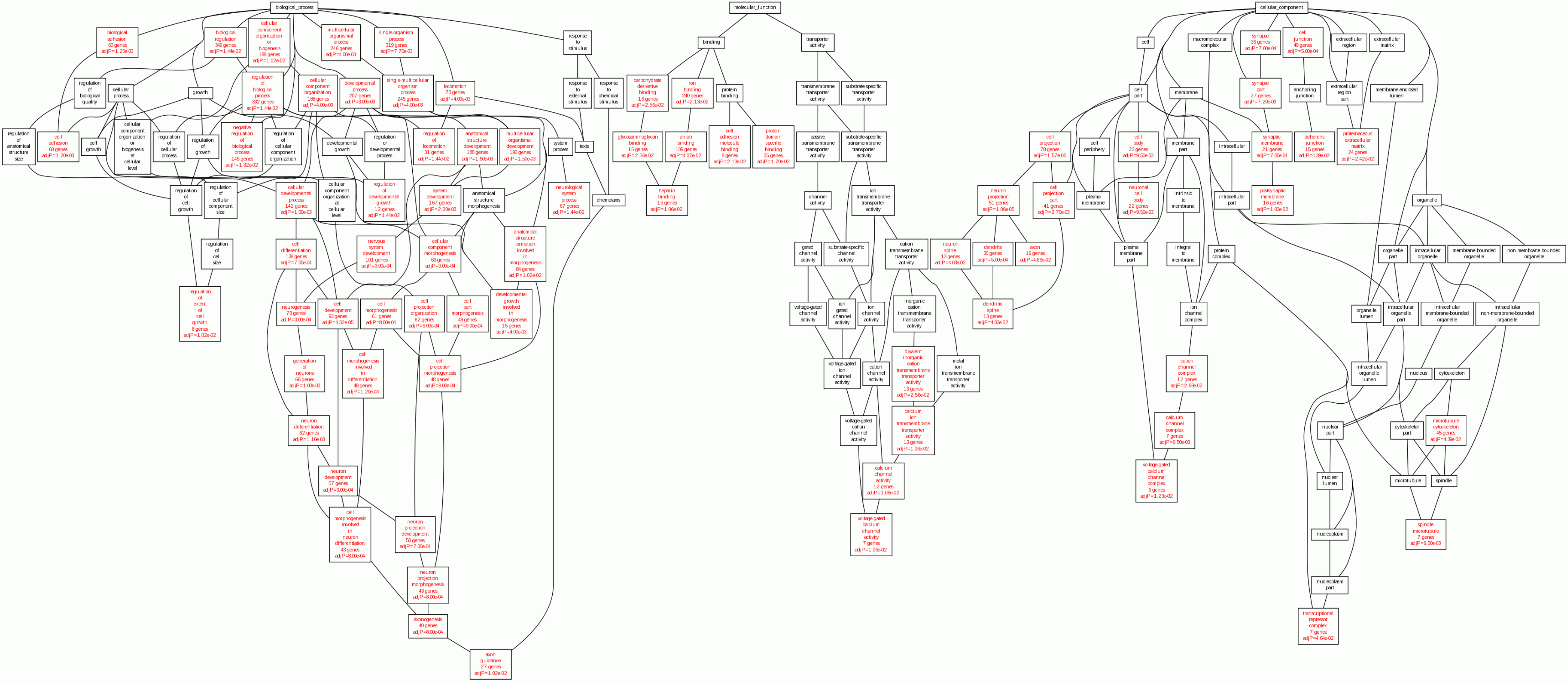

Fig S6. Gene ontology tree showing significant GO term enrichments within the p<0.0001 DMR associated genes. Red indicates significant enrichments (p<0.05 FDR adjusted) with at least 5 genes.

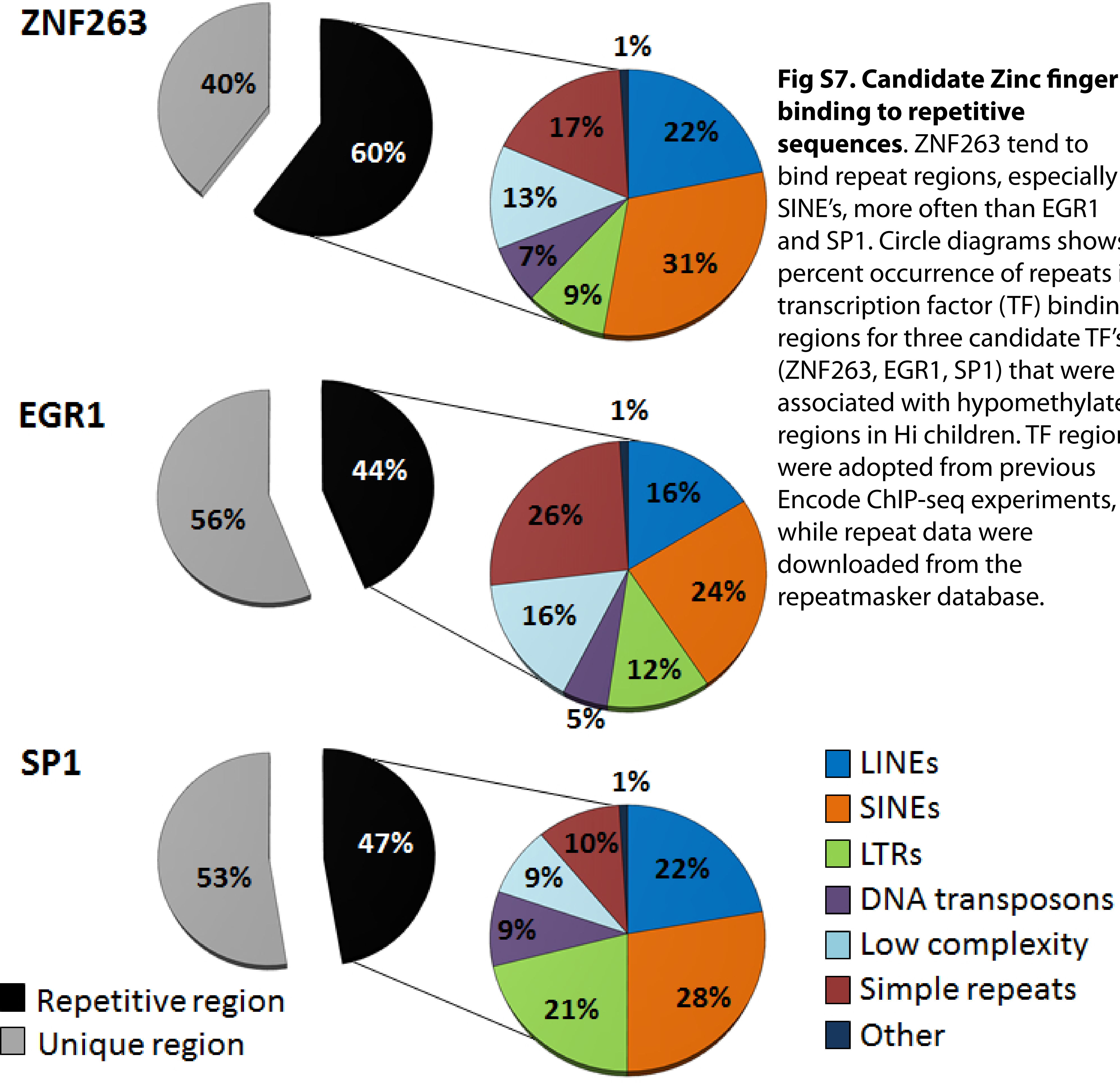

Fig S7. Candidate Zinc finger binding to repetitive sequences. ZNF263 tend to bind repeat regions, especially SINE's, more often than EGR1 and SP1. Circle diagrams shows percent occurrence of repeats in transcription factor (TF) binding regions for three candidate TF's (ZNF263, EGR1, SP1) that were associated with hypomethylated regions in Hi children. TF regions were adopted from previous Encode ChIP-seq experiments, while repeat data were downloaded from the repeatmasker database.

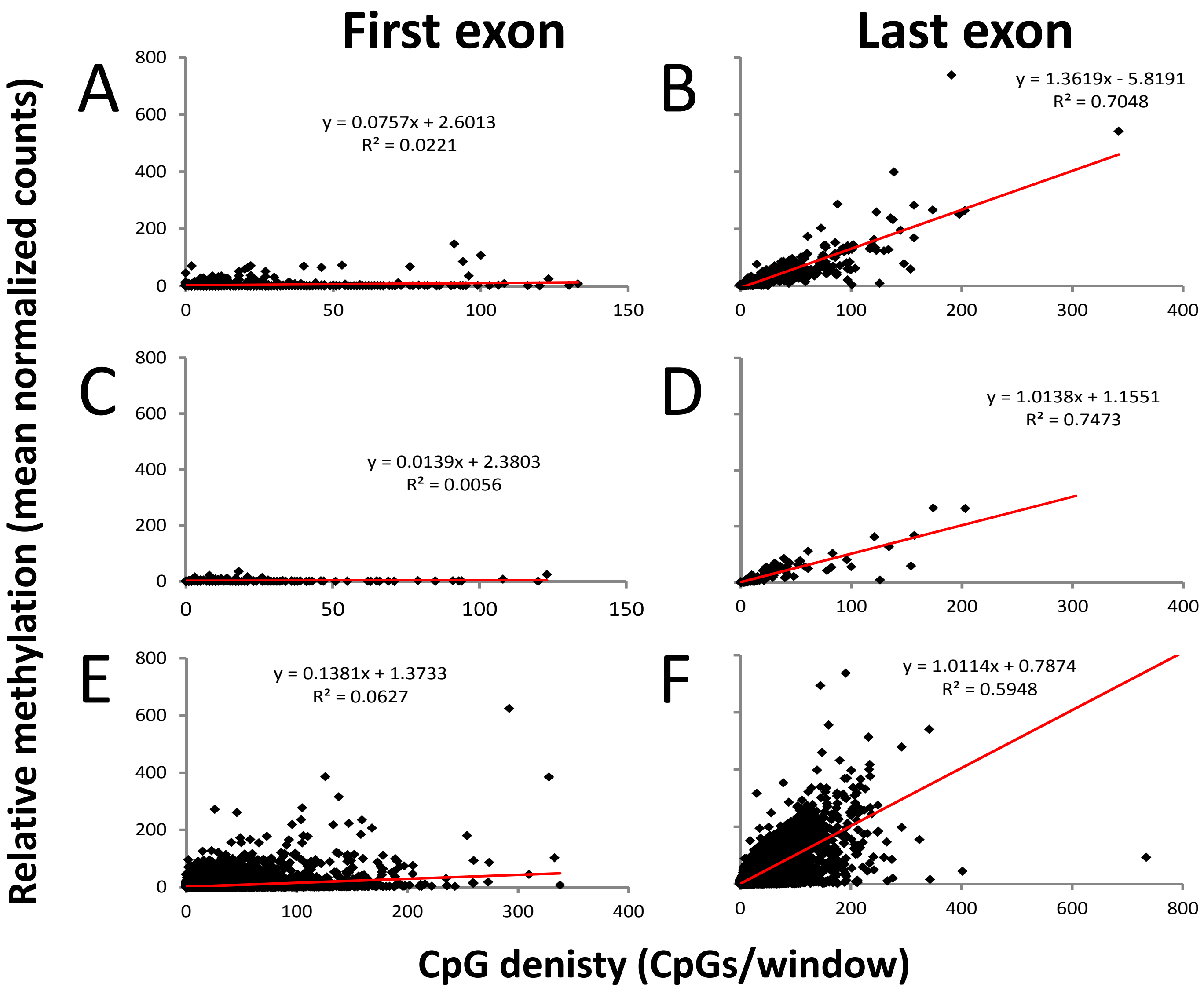

Fig S8. First exon versus last exon methylation relative to number of CpG's. Individual dots represent mean per individual exon across all children (n=48). **A** and **B** plots show genes closest to differentially methylated regions (DMR's, $p<0.0001$) between high and low hair cortisol children. **C** and **D** shows the subset of DMR's associated genes that previously been associated with disease. **E** and **F** shows all first and last exons, genome wide. Included are only genes with at least three exons.

2. Supplemental tables

Table S1. Some demographic and social data of the participating children.

	Cortisol group				
	High		Low		*p-value*
**Gender (n, %):**					
boys	8	42	11	58	0.38
girls	16	55	13	45	
**Birth weight (kg):**					
mean	3.75		3.46		0.11[A]
s.d	0.63		0.61		
**Birth length (cm):**					
mean	51.1		50.6		0.43[A]
s.d	2.2		2.5		
**Normal vaginal delivery (n, %) [B] :**					
yes	22	92	22	96	0.51
no	2	8	1	4	
**Mother born in Sweden (n, %):**					
yes	21	87	21	87	0.67
no	3	13	3	13	
**Father born in Sweden (n, %):**					
yes	22	91	22	91	0.7
no	2	9	2	9	
**Mother educational level (n, %):**					
low	12	50	7	29	0.24
high	12	50	17	71	
**Father educational level (n, %)[B]:**					
low	12	54	10	44	0.46
high	10	46	13	56	
**Housing (n, %)[B]:**					
villa/own house	16	70	19	83	0.49
apartment	7	30	4	17	
**Mother smoker (n, %)[B] (child age 3):**					
yes	2	9	2	9	0.7
no	21	91	21	91	
**Mother smoker (n, %) (child age 5):**					
yes	3	12	1	4	0.61
no	21	88	23	96	
**Mother report experience of serious life events (n, %) (birth):**					
yes	9	37	10	42	0.77
no	15	63	14	58	
**Mother report experience of serious life events (n, %)[B] (child age 3):**					
yes	10	44	5	22	0.21
no	13	56	18	78	
**Mother report good social support for her and her child (n, %)[B] (child age 5):**					
yes	19	83	21	95	0.35
no	4	17	1	5	
**Mother report a safe life situation (n, %)[B] (child age 5):**					
yes	21	88	22	100	0.24
no	3	12	0	0	

All p-values chi^2, except [A] = Anova

[B] At least one non-responders/drop-outs on the question

Table S2. Summary of the main bioinformatics tools used.

	Tool	Purpose	Version	Special settings [A]
Sequencing alignment				
	CASAVA	Demultiplex, Conversion	1.8.2	
	BWA	Alignment to hg19	0.6.2	
	Picard	Sorting, Removal PCR-duplicates	1.69	
Genetic association				
	GATK	SNP-calling	3.3	
	EPACTS	Genetic association	3.2.6	
Methylation analysis				
	MEDIPS	Quality check	1.16.0	
	GenomicRanges	Tiling genomic windows	1.18.4	300 bp
	EDASeq	Normalization	2.0.0	Full quantile
	edgeR	DMR analysis	3.8.5	BH, tagwise disp
Functional analysis				
	WebGestalt	Gene ontology, Pathway analysis	5/20/2014	
	GenometriCorr	Associations in genomic space	1.1.10	10 000 permutations
Sequence analysis				
	MEME	Consensus motif analysis	4.9.1[B]	
	TOMTOM	Consensus motif to transcription factor motif	4.9.1[B]	See Materials and Methods
	FIMO	Consensus motif enrichment in DMR's (+/- 300 bp)	4.9.1[B]	

[A] If not stated otherwise, default settings were used.

[B] Tools are parts of the MEME Suite and share the same version number.

Table S3. Differentially methylated regions in the whole genome analysis

				Genomic location (bp)		
Closest gene	*log2 FC*	*PValue*	*FDR*	*chromosome*	*start*	*end*
PRDM14	-1.37	0.000000002	0.0059	8	70976101	70976400
PPP6R2	0.48	0.000000041	0.0332	22	50870401	50870700
NEK4	-0.73	0.000000056	0.0332	3	52758001	52758300
USP28	0.58	0.000000056	0.0332	11	113710501	113710800
POFUT2	-0.63	0.000000061	0.0332	21	46696501	46696800
CACNA1S	-0.78	0.000000083	0.0332	1	201006301	201006600
ABLIM2	-0.64	0.000000087	0.0332	4	8095801	8096100
GABBR2	-0.87	0.000000089	0.0332	9	101221201	101221500
NBPF3	-1.18	0.000000112	0.0374	1	21752401	21752700
FAM153B	-0.90	0.000000163	0.0488	5	175528501	175528800
FAM49A	-1.02	0.000000272	0.0742	2	16419901	16420200
KCNT2	-1.15	0.000000388	0.0809	1	195754201	195754500
FMO9P	-1.35	0.000000400	0.0809	1	166579801	166580100
LOC101448202	-0.69	0.000000405	0.0809	9	137713501	137713800
TP53BP2	-1.07	0.000000441	0.0809	1	224145001	224145300
THBS2	-1.30	0.000000458	0.0809	6	169665001	169665300
AEBP2	0.67	0.000000459	0.0809	12	19655701	19656000
TULP1	-0.55	0.000000514	0.0856	6	35468701	35469000
CETN1	0.80	0.000000679	0.1035	18	584101	584400
PDE10A	-0.46	0.000000729	0.1035	6	166100701	166101000
AGAP1	-0.40	0.000000759	0.1035	2	236645101	236645400
LINC00595	-0.89	0.000000817	0.1035	10	80097901	80098200
NUP153	0.84	0.000000825	0.1035	6	17620201	17620500
UXS1	-0.96	0.000000828	0.1035	2	106830601	106830900
CDH6	1.03	0.000001068	0.1222	5	31088701	31089000
LRRN1	1.36	0.000001075	0.1222	3	3779701	3780000
CACNA1I	-0.82	0.000001171	0.1222	22	40052101	40052400
MIAT	-0.81	0.000001215	0.1222	22	27150601	27150900
CCDC30	1.10	0.000001216	0.1222	1	42985801	42986100
RRAGC	-1.08	0.000001241	0.1222	1	39186601	39186900
PHAX	1.21	0.000001263	0.1222	5	125949901	125950200
STRN	0.85	0.000001340	0.1257	2	37155301	37155600
TSNARE1	-0.81	0.000001439	0.1308	8	143377501	143377800
SLC45A2	-0.98	0.000001591	0.1333	5	33981001	33981300
STEAP4	-1.09	0.000001666	0.1333	7	87940201	87940500
KRTAP3-2	-0.89	0.000001750	0.1333	17	39156001	39156300
IDE	0.50	0.000001755	0.1333	10	94203001	94203300
NAT1	-1.14	0.000001831	0.1333	8	18141001	18141300
EMP2	0.73	0.000001843	0.1333	16	10610401	10610700
PCM1	0.96	0.000001860	0.1333	8	17805901	17806200
PRRG4	-1.11	0.000001863	0.1333	11	32893501	32893800
MCF2L-AS1	-0.56	0.000001959	0.1333	13	113618401	113618700
SLC12A8	-0.91	0.000001991	0.1333	3	124907401	124907700
ATP11C	-1.18	0.000001995	0.1333	X	138954601	138954900
PER3	-0.85	0.000002011	0.1333	1	7887901	7888200
CTNND2	1.15	0.000002046	0.1333	5	10884301	10884600
IMMP2L	-1.05	0.000002089	0.1333	7	110298601	110298900
RALGAPA2	-0.75	0.000002147	0.1342	20	20388001	20388300
USP17L11	-0.60	0.000002221	0.1360	4	9250501	9250800
CKAP2L	-1.02	0.000002293	0.1376	2	113498401	113498700
CHMP2A	0.68	0.000002351	0.1383	19	59063101	59063400
ALOX5AP	0.88	0.000002486	0.1434	13	31322101	31322400
CNTN5	-0.95	0.000002547	0.1442	11	99544201	99544500
PSMD7	0.74	0.000002838	0.1577	16	74312401	74312700
TADA2B	-0.89	0.000002902	0.1583	4	7046701	7047000
SNORD114-31	1.09	0.000003040	0.1616	14	101470801	101471100
KLHL1	-0.95	0.000003081	0.1616	13	70675801	70676100
LINC00939	-0.50	0.000003210	0.1616	12	126466801	126467100
CDK1	0.96	0.000003256	0.1616	10	62558401	62558700
UACA	1.03	0.000003385	0.1616	15	71057101	71057400
NR2C1	0.71	0.000003472	0.1616	12	95431201	95431500
OPN4	-1.07	0.000003516	0.1616	10	88419901	88420200
BPIFB1	0.79	0.000003582	0.1616	20	31902301	31902600
AGAP9	-1.13	0.000003585	0.1616	10	47192401	47192700
KATNB1	0.40	0.000003643	0.1616	16	57767701	57768000
SEC63	0.99	0.000003724	0.1616	6	108189001	108189300

PBX4	-0.39	0.000003738	0.1616	19	19699801	19700100
KCNMB2	0.98	0.000003906	0.1616	3	178596301	178596600
FLJ33360	-1.25	0.000003920	0.1616	5	6219301	6219600
TPD52	1.16	0.000003925	0.1616	8	80982601	80982900
MIR4278	0.80	0.000003993	0.1616	5	6899401	6899700
HS3ST4	-1.09	0.000003995	0.1616	16	26148901	26149200
FAM49A	-0.92	0.000004018	0.1616	2	16419601	16419900
CLCN4	-0.59	0.000004090	0.1616	X	10174501	10174800
LINC00948	-1.06	0.000004112	0.1616	10	61507201	61507500
VTCN1	-0.89	0.000004144	0.1616	1	117783901	117784200
ERRFI1	0.82	0.000004205	0.1616	1	8141701	8142000
RPRD1B	0.77	0.000004246	0.1616	20	36717001	36717300
C10orf35	0.93	0.000004287	0.1616	10	71445001	71445300
SOD2	-0.64	0.000004321	0.1616	6	160032301	160032600
GPC5	-0.86	0.000004364	0.1616	13	92934301	92934600
LMO7	-0.80	0.000004577	0.1652	13	76262101	76262400
TMEM18	-0.79	0.000004713	0.1652	2	611401	611700
LINC00838	1.09	0.000004727	0.1652	10	33960901	33961200
CTNNA1	-0.86	0.000004790	0.1652	5	138270601	138270900
ZNF420	0.87	0.000004809	0.1652	19	37530601	37530900
TPBG	0.95	0.000004870	0.1652	6	83169601	83169900
CDK1	1.04	0.000004905	0.1652	10	62558701	62559000
BRCA1	-0.81	0.000004950	0.1652	17	41241601	41241900
APBB2	0.78	0.000004956	0.1652	4	41043901	41044200
KRT18P55	-0.54	0.000005189	0.1702	17	26631001	26631300
ATG7	-1.05	0.000005345	0.1702	3	11546401	11546700
FAM78A	-0.88	0.000005357	0.1702	9	134146501	134146800
KLF5	0.79	0.000005410	0.1702	13	73949701	73950000
USP17L25	-0.67	0.000005424	0.1702	4	9327901	9328200
KCNF1	-0.95	0.000005550	0.1702	2	11064001	11064300
LINC00276	-0.76	0.000005555	0.1702	2	14517901	14518200
SERP1	0.92	0.000005573	0.1702	3	150244201	150244500
RP11-770E5.1	-1.05	0.000005623	0.1702	8	49251301	49251600
GPR68	-0.89	0.000005717	0.1702	14	91706701	91707000
MIR2054	1.16	0.000005777	0.1702	4	126646801	126647100
SPATA31C1	-0.71	0.000005809	0.1702	9	90558901	90559200
DKFZp566F0947	-0.85	0.000005865	0.1702	19	31465201	31465500
LOC729159	0.82	0.000005943	0.1702	16	60557701	60558000
LOC101929034	-0.78	0.000005963	0.1702	5	1843501	1843800
LOC595101	0.69	0.000006015	0.1702	16	30353401	30353700
ZNF664-FAM101A	-0.88	0.000006210	0.1709	12	124617601	124617900
IGFBP2	1.45	0.000006220	0.1709	2	217442701	217443000
KLHL29	-0.87	0.000006252	0.1709	2	23171401	23171700
SUMO1	1.03	0.000006266	0.1709	2	203079901	203080200
P2RY12	-0.65	0.000006326	0.1710	3	151092301	151092600
APPBP2	-0.93	0.000006403	0.1715	17	58586401	58586700
ATF7IP2	1.03	0.000006515	0.1723	16	10445401	10445700
LOC344967	-0.74	0.000006547	0.1723	4	40043701	40044000
RBM33	-1.05	0.000006767	0.1754	7	155549101	155549400
SH2D3A	-1.00	0.000006781	0.1754	19	6759601	6759900
LINC00210	0.50	0.000006937	0.1779	1	218068201	218068500
SEMA4D	0.75	0.000007019	0.1784	9	91988401	91988700
FBN2	-1.03	0.000007128	0.1797	5	127683001	127683300
ERG	-0.57	0.000007220	0.1805	21	40068001	40068300
HAS2	0.76	0.000007381	0.1819	8	122345701	122346000
ACN9	-1.16	0.000007398	0.1819	7	96839401	96839700
ELOVL6	1.20	0.000007494	0.1828	4	111247501	111247800
EXOG	-0.72	0.000007628	0.1845	3	38547001	38547300
SERINC5	-0.74	0.000007839	0.1866	5	79443901	79444200
TPSD1	0.72	0.000007899	0.1866	16	1300501	1300800
COL5A3	-0.75	0.000007900	0.1866	19	10107901	10108200
GNB2L1	-0.98	0.000007976	0.1869	5	180665101	180665400
THSD4	-0.72	0.000008116	0.1888	15	72042901	72043200
RAB11FIP4	-0.72	0.000008238	0.1899	17	29741701	29742000
TGFA	0.70	0.000008316	0.1899	2	70665301	70665600
CACNB2	0.57	0.000008357	0.1899	10	18738301	18738600
FAM220A	0.47	0.000008544	0.1910	7	6346501	6346800
MESP1	0.72	0.000008570	0.1910	15	90298201	90298500
GPM6A	0.99	0.000008647	0.1910	4	176692501	176692800
TRIM61	0.62	0.000008668	0.1910	4	165914101	165914400

LOC389033	0.65	0.000008787	0.1910	2	130184701	130185000
FOXP1	-0.89	0.000008837	0.1910	3	70982701	70983000
PICK1	-0.98	0.000008848	0.1910	22	38464201	38464500
ST5	0.75	0.000009052	0.1940	11	8833201	8833500
MIR205HG	-0.89	0.000009188	0.1946	1	209261401	209261700
SEMA5A	-1.08	0.000009210	0.1946	5	9108301	9108600
SYTL4	-1.08	0.000009460	0.1985	X	99982801	99983100
BMS1	-0.54	0.000009666	0.2014	10	43368601	43368900
STAM2	-0.96	0.000009739	0.2015	2	153029701	153030000
TPGS1	0.90	0.000010166	0.2077	19	519601	519900
MMP24	0.40	0.000010177	0.2077	20	33856501	33856800
LOC101929681	-0.90	0.000010349	0.2089	5	29980201	29980500
PRDM6	-1.02	0.000010383	0.2089	5	122446801	122447100
RP4-723E3.1	-1.08	0.000010452	0.2089	20	51290701	51291000
IDUA	-0.88	0.000010515	0.2089	4	1001401	1001700
MIR548N	0.64	0.000010597	0.2091	2	179381701	179382000
TLE4	0.80	0.000010868	0.2124	9	82159501	82159800
RGS14	-0.87	0.000010902	0.2124	5	176795401	176795700
LOC650293	-1.17	0.000011014	0.2132	4	9054601	9054900
CPN1	0.90	0.000011209	0.2156	10	101790301	101790600
LEF1-AS1	0.54	0.000011411	0.2169	4	109107001	109107300
ATP8A1	0.92	0.000011425	0.2169	4	42539101	42539400
ACSL3	-0.83	0.000011645	0.2175	2	223801201	223801500
MEIS2	-0.92	0.000011803	0.2175	15	37761301	37761600
PSPC1	0.85	0.000011898	0.2175	13	20318701	20319000
HPD	0.50	0.000011969	0.2175	12	122288101	122288400
RHCE	-0.83	0.000011999	0.2175	1	25751701	25752000
RP11-71G7.1	-1.13	0.000012021	0.2175	3	176540701	176541000
LOC100133331	0.63	0.000012185	0.2175	1	332401	332700
C9orf92	-1.22	0.000012245	0.2175	9	16133401	16133700
SLC24A2	-0.84	0.000012307	0.2175	9	19958401	19958700
SCN8A	-0.55	0.000012309	0.2175	12	52187401	52187700
EXOC3L2	-0.83	0.000012397	0.2175	19	45744901	45745200
DMBT1	-0.96	0.000012410	0.2175	10	124305901	124306200
CPA4	0.35	0.000012501	0.2175	7	129932101	129932400
LINC00929	-0.57	0.000012550	0.2175	15	26363701	26364000
FAM21C	0.90	0.000012630	0.2175	10	46281601	46281900
LINC00970	-0.97	0.000012655	0.2175	1	168986101	168986400
GUSBP1	0.82	0.000012687	0.2175	5	21462601	21462900
MIR4634	0.60	0.000012947	0.2204	5	174169501	174169800
FHAD1	-1.05	0.000013006	0.2204	1	15575401	15575700
TMEM41B	-0.59	0.000013160	0.2209	11	9343801	9344100
MED12L	-1.18	0.000013232	0.2209	3	151137901	151138200
IFFO2	0.87	0.000013252	0.2209	1	19288501	19288800
LOC100132831	0.58	0.000013447	0.2222	X	40685401	40685700
TRAFD1	0.69	0.000013477	0.2222	12	112580701	112581000
NGF	-1.55	0.000013719	0.2249	1	116001901	116002200
EMB	-0.99	0.000013822	0.2254	5	49800301	49800600
DCUN1D4	-1.03	0.000014009	0.2264	4	52710301	52710600
SIN3A	0.63	0.000014059	0.2264	15	75724801	75725100
FAM189A1	-0.59	0.000014201	0.2264	15	29668801	29669100
USP17L30	-0.44	0.000014257	0.2264	4	9353101	9353400
SHOX	-0.72	0.000014262	0.2264	X	643801	644100
LYRM4	-0.91	0.000014362	0.2268	6	5244901	5245200
TBC1D3H	-0.84	0.000014594	0.2289	17	34757401	34757700
SLCO3A1	-0.84	0.000014666	0.2289	15	92678701	92679000
DIAPH3	-0.82	0.000014834	0.2289	13	59435701	59436000
LDLRAD3	-0.75	0.000014887	0.2289	11	36147601	36147900
USP17L25	-0.42	0.000014941	0.2289	4	9342601	9342900
MIR138-1	-0.90	0.000014958	0.2289	3	44203501	44203800
SHANK2	-0.66	0.000015029	0.2289	11	70547101	70547400
AP1S3	-0.88	0.000015296	0.2317	2	224630701	224631000
LUCAT1	-0.81	0.000015486	0.2320	5	90534601	90534900
LIPH	-0.71	0.000015512	0.2320	3	185230501	185230800
LARP1	1.05	0.000015623	0.2320	5	154068001	154068300
CELF4	-0.91	0.000015666	0.2320	18	34923301	34923600
MYOZ2	-0.74	0.000015866	0.2320	4	120094501	120094800
SETBP1	1.15	0.000015868	0.2320	18	42414001	42414300
RIMS1	-0.79	0.000015872	0.2320	6	72700501	72700800
TBL1XR1	-0.96	0.000015990	0.2320	3	176895301	176895600

GAREM	0.78	0.000016010	0.2320	18	29897101	29897400
SKA3	-0.66	0.000016273	0.2341	13	21744301	21744600
PP2D1	0.65	0.000016322	0.2341	3	20040601	20040900
PRNP	0.42	0.000016436	0.2341	20	4569601	4569900
REG3G	-1.04	0.000016467	0.2341	2	79252801	79253100
CRBN	-0.87	0.000016695	0.2363	3	3326701	3327000
CACHD1	-1.29	0.000016917	0.2375	1	65033401	65033700
NPLOC4	-0.70	0.000016955	0.2375	17	79524901	79525200
ANKRD20A9P	-0.63	0.000017024	0.2375	13	19456501	19456800
OCIAD2	0.96	0.000017153	0.2380	4	48915001	48915300
FLNB	-0.94	0.000017244	0.2380	3	58113901	58114200
PANX2	0.46	0.000017291	0.2380	22	50612701	50613000
NAAA	-0.92	0.000017439	0.2389	4	76865701	76866000
MKI67	-0.59	0.000017627	0.2396	10	130079401	130079700
SHCBP1L	-0.54	0.000017915	0.2396	1	182913001	182913300
EPC1	-0.60	0.000017963	0.2396	10	32591101	32591400
RHOT1	-1.05	0.000017996	0.2396	17	30529201	30529500
RPS6KA2	-0.63	0.000018059	0.2396	6	166972201	166972500
COA1	0.87	0.000018089	0.2396	7	43703101	43703400
ZNF566	0.53	0.000018160	0.2396	19	36931501	36931800
GML	-0.76	0.000018266	0.2396	8	143915401	143915700
FCRL5	0.68	0.000018300	0.2396	1	157342801	157343100
SPTY2D1	-0.94	0.000018310	0.2396	11	18686101	18686400
WDR59	0.59	0.000018369	0.2396	16	74961901	74962200
SUPT6H	0.34	0.000018525	0.2398	17	27018901	27019200
C8orf59	0.53	0.000018545	0.2398	8	86136301	86136600
ALMS1	-1.01	0.000018736	0.2404	2	73633801	73634100
TMEM72	-0.93	0.000018753	0.2404	10	45429001	45429300
RASSF9	-0.70	0.000018885	0.2411	12	85991701	85992000
MAN2B2	-1.18	0.000019188	0.2439	4	6572101	6572400
MYH16	-0.92	0.000019320	0.2441	7	98907601	98907900
RBP1	-0.99	0.000019362	0.2441	3	139239001	139239300
ROBO1	0.82	0.000019645	0.2447	3	79023301	79023600
ZNF607	0.43	0.000019704	0.2447	19	38195401	38195700
GRK4	-0.97	0.000019803	0.2447	4	3024901	3025200
RCC1	0.94	0.000019804	0.2447	1	28837801	28838100
RP3-323P13.2	0.62	0.000019821	0.2447	6	134126701	134127000
LYRM9	0.75	0.000020098	0.2466	17	26246101	26246400
FBLN7	-0.86	0.000020163	0.2466	2	112931401	112931700
LRRN1	-1.06	0.000020322	0.2466	3	3985801	3986100
MMD2	-0.85	0.000020343	0.2466	7	4931701	4932000
ATP2B3	-0.85	0.000020465	0.2466	X	152806201	152806500
COL4A1	-0.54	0.000020496	0.2466	13	110879701	110880000
AK9	-0.59	0.000020673	0.2466	6	109818301	109818600
USP17L17	-0.34	0.000020707	0.2466	4	9242401	9242700
FAM181B	-0.52	0.000020711	0.2466	11	82428901	82429200
PRKAA1	0.77	0.000020998	0.2469	5	40793701	40794000
MME	-0.83	0.000021015	0.2469	3	154518901	154519200
OGDH	0.45	0.000021191	0.2469	7	44744701	44745000
SEC61B	-1.07	0.000021251	0.2469	9	102034201	102034500
NAA15	-0.80	0.000021294	0.2469	4	140262301	140262600
SAT1	-0.87	0.000021307	0.2469	X	23784001	23784300
RP11-347D21.3	-0.57	0.000021395	0.2469	20	46750201	46750500
AJAP1	-1.12	0.000021401	0.2469	1	5202601	5202900
DNAJC15	-0.59	0.000021494	0.2471	13	43633801	43634100
ARHGEF5	-0.86	0.000021786	0.2477	7	144070801	144071100
LINC01012	-0.87	0.000021799	0.2477	6	27641101	27641400
SEMA7A	-0.45	0.000021802	0.2477	15	74710201	74710500
RCSD1	0.64	0.000021911	0.2481	1	167575501	167575800
FAM19A1	0.98	0.000022069	0.2487	3	68126701	68127000
ERVV-2	0.72	0.000022188	0.2487	19	53539201	53539500
ST3GAL6	0.72	0.000022218	0.2487	3	98504701	98505000
FER	1.22	0.000022480	0.2490	5	108163501	108163800
HS3ST4	-0.93	0.000022574	0.2490	16	25996801	25997100
DCUN1D2	-0.91	0.000022618	0.2490	13	114124501	114124800
INTU	0.76	0.000022637	0.2490	4	127881601	127881900
EYS	-0.87	0.000022725	0.2490	6	64522801	64523100
PTPRD	1.04	0.000022790	0.2490	9	8517001	8517300
RSPO2	1.07	0.000023034	0.2490	8	108963901	108964200
CHST13	-0.74	0.000023069	0.2490	3	126254401	126254700

RP11-274B18.2	-0.88	0.000023166	0.2490	9	71237701	71238000
CAMK4	1.05	0.000023205	0.2490	5	110541601	110541900
SELE	-1.03	0.000023398	0.2490	1	169696201	169696500
CA5A	0.50	0.000023520	0.2490	16	87956701	87957000
LYZL4	1.11	0.000023601	0.2490	3	42431101	42431400
RNF41	-0.60	0.000023630	0.2490	12	56594401	56594700
LTBP2	0.57	0.000023703	0.2490	14	75009301	75009600
ZNF618	-1.01	0.000023711	0.2490	9	116771401	116771700
PRDM7	-1.03	0.000023724	0.2490	16	90171601	90171900
CTNNA3	-0.79	0.000023736	0.2490	10	67641301	67641600
MLLT10P1	0.85	0.000023968	0.2495	20	29651101	29651400
AC131097.3	0.75	0.000024089	0.2495	2	243003601	243003900
LSP1P3	-0.94	0.000024221	0.2495	5	28830901	28831200
NCOA1	0.48	0.000024233	0.2495	2	24711601	24711900
ITM2B	0.80	0.000024535	0.2495	13	48743401	48743700
RP11-638F5.1	-0.61	0.000024687	0.2495	12	131677201	131677500
BOK-AS1	-0.82	0.000024794	0.2495	2	242490301	242490600
TMCO6	-1.02	0.000024867	0.2495	5	140022601	140022900
KIF11	0.86	0.000024917	0.2495	10	94416301	94416600
GPR39	0.91	0.000024966	0.2495	2	133312201	133312500
GFRA1	-0.67	0.000024998	0.2495	10	117872401	117872700
GUCA1C	0.90	0.000025045	0.2495	3	108647401	108647700
BCL6	1.00	0.000025077	0.2495	3	187635001	187635300
ST8SIA5	-0.86	0.000025092	0.2495	18	44325601	44325900
RNF220	0.45	0.000025106	0.2495	1	45086401	45086700
CDH22	-0.88	0.000025112	0.2495	20	44839501	44839800
CDH23	-0.66	0.000025692	0.2540	10	73153501	73153800
SLC8A1-AS1	-0.99	0.000025738	0.2540	2	40242901	40243200
PHOX2B	-0.96	0.000025849	0.2542	4	41742001	41742300
ENOPH1	0.38	0.000026255	0.2553	4	83365201	83365500
INSC	-0.77	0.000026478	0.2553	11	15566101	15566400
EMP1	-0.92	0.000026610	0.2553	12	13338001	13338300
ABCB9	-0.87	0.000026710	0.2553	12	123418501	123418800
ALPK2	0.87	0.000026714	0.2553	18	56302501	56302800
LOC101448202	-0.92	0.000026859	0.2553	9	137713801	137714100
DEFB131	-1.11	0.000026884	0.2553	4	9443401	9443700
ABLIM2	-0.41	0.000027030	0.2553	4	8096101	8096400
PLXNC1	-1.13	0.000027057	0.2553	12	94689301	94689600
ELAVL2	0.60	0.000027057	0.2553	9	23663401	23663700
RNF6	-0.63	0.000027155	0.2553	13	26729401	26729700
SFRP1	-0.78	0.000027369	0.2553	8	40987201	40987500
MCHR2	-0.75	0.000027625	0.2553	6	100423501	100423800
MYB	0.80	0.000027639	0.2553	6	135499201	135499500
LINC00323	-0.71	0.000027639	0.2553	21	42425101	42425400
ARHGAP1	-0.86	0.000027661	0.2553	11	46698901	46699200
RTN4	-0.52	0.000027714	0.2553	2	55337701	55338000
LOC100652824	-0.77	0.000027728	0.2553	2	202951801	202952100
KLHL29	-1.24	0.000027856	0.2553	2	23871301	23871600
TDRD3	0.50	0.000027869	0.2553	13	60891301	60891600
SCAPER	-1.02	0.000027882	0.2553	15	76857001	76857300
AACS	0.89	0.000027900	0.2553	12	125636701	125637000
AC007966.1	-0.90	0.000027912	0.2553	2	186357301	186357600
SLC14A2	-0.83	0.000028049	0.2553	18	43189201	43189500
USP17L15	-0.45	0.000028078	0.2553	4	9237601	9237900
LINC00948	-0.90	0.000028317	0.2556	10	61507501	61507800
UNC5D	-0.67	0.000028354	0.2556	8	35696701	35697000
VRK1	0.72	0.000028375	0.2556	14	97248601	97248900
ZNF207	0.55	0.000028453	0.2556	17	30712801	30713100
ZRANB3	1.37	0.000028567	0.2558	2	136260001	136260300
CCDC7	-1.02	0.000028818	0.2560	10	33075601	33075900
SOD2	0.71	0.000028987	0.2560	6	159903601	159903900
MYO1E	0.90	0.000029066	0.2560	15	59539201	59539500
CDH4	-0.85	0.000029150	0.2560	20	59969701	59970000
ARNTL	-0.77	0.000029163	0.2560	11	13200901	13201200
STIM2	1.19	0.000029216	0.2560	4	27266701	27267000
METTL11B	0.64	0.000029306	0.2560	1	170133001	170133300
MIR548H3	-0.83	0.000029335	0.2560	9	78211801	78212100
ARL4C	-1.10	0.000029383	0.2560	2	235381801	235382100
DAGLB	0.33	0.000029493	0.2560	7	6491701	6492000
ZBTB16	-0.90	0.000029523	0.2560	11	114015001	114015300

MIR3659	-0.81	0.000029847	0.2580	1	38585401	38585700
MIR4283-2	-0.61	0.000030094	0.2590	7	57015001	57015300
L3MBTL4	0.85	0.000030134	0.2590	18	6462601	6462900
SPIN1	-0.51	0.000030439	0.2599	9	91116601	91116900
NPHP3-ACAD11	0.55	0.000030465	0.2599	3	132305701	132306000
SLC24A4	-0.40	0.000030496	0.2599	14	92718901	92719200
SYBU	-1.19	0.000030673	0.2604	8	110703301	110703600
AGAP1	-0.79	0.000030730	0.2604	2	236745901	236746200
FAM220A	0.34	0.000031161	0.2630	7	6346801	6347100
USP17L25	-0.35	0.000031213	0.2630	4	9337801	9338100
DEPDC1	0.49	0.000031561	0.2645	1	69127201	69127500
LPP	-0.55	0.000031608	0.2645	3	187878001	187878300
CAPZA3	-0.89	0.000031683	0.2645	12	18906301	18906600
PIP5K1C	0.39	0.000032006	0.2645	19	3639001	3639300
LRBA	1.03	0.000032017	0.2645	4	151751701	151752000
NMNAT3	-0.69	0.000032030	0.2645	3	139509301	139509600
CHRNA4	0.46	0.000032136	0.2645	20	62014201	62014500
ARL6IP6	-0.94	0.000032495	0.2645	2	153647101	153647400
B3GALT1	-0.88	0.000032511	0.2645	2	168584401	168584700
QRFPR	0.88	0.000032555	0.2645	4	122203501	122203800
C2orf54	0.41	0.000032559	0.2645	2	241829101	241829400
GALNT14	0.34	0.000032587	0.2645	2	31120201	31120500
RASAL2	-0.98	0.000032623	0.2645	1	178338001	178338300
MIR563	0.69	0.000032689	0.2645	3	15921001	15921300
LOC102577424	0.71	0.000032871	0.2645	5	176157901	176158200
ARHGAP36	-0.92	0.000033002	0.2645	X	130298701	130299000
ADAM22	-0.86	0.000033065	0.2645	7	87640801	87641100
FAM25G	-0.91	0.000033071	0.2645	10	47179201	47179500
DENND1A	0.80	0.000033236	0.2645	9	126155701	126156000
CLEC14A	-0.50	0.000033242	0.2645	14	38956801	38957100
TAF4B	0.52	0.000033244	0.2645	18	23908801	23909100
RARRES1	-0.81	0.000033673	0.2670	3	158438101	158438400
AL109763.2	-0.87	0.000033775	0.2670	21	19866001	19866300
MCU	0.72	0.000033822	0.2670	10	74457301	74457600
KIF3B	0.89	0.000034161	0.2687	20	30886201	30886500
SNCG	-0.53	0.000034215	0.2687	10	88721401	88721700
CERCAM	0.77	0.000034606	0.2706	9	131199001	131199300
ADAMTS2	-0.41	0.000034642	0.2706	5	178789201	178789500
BAIAP2L2	-0.77	0.000034804	0.2710	22	38498101	38498400
ADCYAP1R1	0.99	0.000034956	0.2710	7	31169401	31169700
CRTC2	-1.33	0.000034963	0.2710	1	153920401	153920700
RBM42	-0.94	0.000035146	0.2717	19	36121801	36122100
ACTR3BP2	1.09	0.000035331	0.2718	2	92202301	92202600
ARHGEF10	-0.97	0.000035340	0.2718	8	1823701	1824000
APOD	0.55	0.000035423	0.2718	3	195296401	195296700
DNAH8	-0.86	0.000036000	0.2755	6	38851801	38852100
LITAF	-0.83	0.000036332	0.2763	16	11613901	11614200
RP11-69H14.6	0.87	0.000036344	0.2763	15	22314601	22314900
RFC1	1.27	0.000036557	0.2763	4	39359101	39359400
RBMX	0.63	0.000036566	0.2763	X	136023601	136023900
RAB2A	0.72	0.000036809	0.2763	8	61516501	61516800
DMAP1	-0.75	0.000036910	0.2763	1	44682001	44682300
PDE8B	-1.00	0.000036937	0.2763	5	76615201	76615500
TRIM16L	0.55	0.000036976	0.2763	17	18607201	18607500
MSN	0.84	0.000037103	0.2763	X	65087101	65087400
PLD5	0.41	0.000037244	0.2763	1	242639401	242639700
RP11-265P11.2	-0.48	0.000037297	0.2763	X	39435601	39435900
RPTOR	-0.51	0.000037359	0.2763	17	78594301	78594600
NKIRAS1	0.55	0.000037379	0.2763	3	23937901	23938200
KIT	-0.85	0.000037438	0.2763	4	55446301	55446600
RXRA	-0.59	0.000037486	0.2763	9	137142601	137142900
BDH1	-1.05	0.000037717	0.2764	3	197314501	197314800
KHDRBS3	-0.77	0.000037811	0.2764	8	136611901	136612200
KAT5	-0.65	0.000037865	0.2764	11	65480701	65481000
SND1	-0.95	0.000037871	0.2764	7	127595701	127596000
REEP3	0.79	0.000038269	0.2771	10	65621701	65622000
MTF2	0.72	0.000038302	0.2771	1	93555901	93556200
AASDH	0.60	0.000038318	0.2771	4	57236101	57236400
LINC01029	-0.92	0.000038333	0.2771	18	75636901	75637200
SPATA16	-0.84	0.000038476	0.2774	3	172875601	172875900

PARP11	-1.01	0.000038563	0.2774	12	3903001	3903300
LRIG1	-0.96	0.000038843	0.2786	3	66789001	66789300
ACKR3	-0.54	0.000039083	0.2786	2	237501301	237501600
GRIN3A	-0.96	0.000039093	0.2786	9	104559301	104559600
ZNF254	0.97	0.000039096	0.2786	19	24326701	24327000
PPP2R3B	0.65	0.000039384	0.2794	Y	404701	405000
RP11-730N24.1	1.01	0.000039398	0.2794	5	24892201	24892500
CCDC71L	-0.92	0.000039782	0.2815	7	106164001	106164300
TPI1	-0.76	0.000040118	0.2827	12	6979201	6979500
UBE2N	0.77	0.000040138	0.2827	12	93820801	93821100
ADAMTS18	0.69	0.000040324	0.2833	16	77453701	77454000
SLC5A1	-0.85	0.000040465	0.2836	22	32452201	32452500
TMEM232	-0.97	0.000040919	0.2847	5	109856101	109856400
SLC25A26	-0.91	0.000040924	0.2847	3	66349801	66350100
OAT	0.75	0.000040945	0.2847	10	126040501	126040800
MST1R	-1.10	0.000041001	0.2847	3	49924501	49924800
SH3BP2	-0.55	0.000041330	0.2861	4	2808601	2808900
STK32B	-0.89	0.000041473	0.2861	4	5487301	5487600
CDK19	-0.97	0.000041551	0.2861	6	111129301	111129600
HGS	0.30	0.000041630	0.2861	17	79657501	79657800
NAIP	-0.87	0.000041920	0.2861	5	70270801	70271100
AFAP1	0.93	0.000042090	0.2861	4	7793701	7794000
ELP3	0.53	0.000042094	0.2861	8	28107301	28107600
CALB2	-0.82	0.000042113	0.2861	16	71400901	71401200
BTBD9	-0.92	0.000042230	0.2861	6	38382301	38382600
ADAMTSL1	0.54	0.000042261	0.2861	9	18567001	18567300
OTX2	0.73	0.000042392	0.2861	14	57201901	57202200
LINC00381	-0.93	0.000042446	0.2861	13	75034501	75034800
TCF7	-0.94	0.000042540	0.2861	5	133446301	133446600
FOXK1	0.79	0.000042832	0.2861	7	4706701	4707000
SMAD9	0.48	0.000042992	0.2861	13	37476601	37476900
CTR9	-0.95	0.000043045	0.2861	11	10803001	10803300
C1orf100	0.69	0.000043197	0.2861	1	244378501	244378800
PTCHD3	-0.67	0.000043257	0.2861	10	27676201	27676500
CHMP1A	-0.57	0.000043466	0.2861	16	89717701	89718000
TPO	0.89	0.000043475	0.2861	2	1531201	1531500
ACOT13	0.97	0.000043482	0.2861	6	24693601	24693900
IGF1R	0.75	0.000043602	0.2861	15	99423301	99423600
GLRX3	-0.73	0.000043628	0.2861	10	132052801	132053100
CCDC60	0.93	0.000043637	0.2861	12	119951401	119951700
FLJ45079	-0.92	0.000043834	0.2861	17	75913201	75913500
MIR4478	-0.91	0.000043946	0.2861	9	124878901	124879200
FBXO47	-0.82	0.000043990	0.2861	17	37110301	37110600
USP17L25	-0.59	0.000044001	0.2861	4	9351901	9352200
C4orf51	-0.78	0.000044029	0.2861	4	146605201	146605500
ZNF354B	-0.95	0.000044274	0.2861	5	178268101	178268400
BCKDHB	0.41	0.000044305	0.2861	6	81197101	81197400
RBM42	-0.85	0.000044306	0.2861	19	36122101	36122400
ACOT6	0.75	0.000044399	0.2861	14	74086501	74086800
SAMD4A	0.61	0.000044440	0.2861	14	55142401	55142700
GATS	-0.90	0.000044617	0.2866	7	99853801	99854100
ELF5	-1.34	0.000044825	0.2869	11	34514701	34515000
CDH6	-1.70	0.000044869	0.2869	5	30495601	30495900
ERCC5	1.00	0.000044993	0.2869	13	103500301	103500600
CYTL1	-0.77	0.000045037	0.2869	4	4991101	4991400
NRP2	0.29	0.000045143	0.2869	2	206562301	206562600
LINC00709	0.61	0.000045325	0.2874	10	9240601	9240900
PTPRU	-0.85	0.000045543	0.2874	1	29587201	29587500
ARHGEF26-AS1	-1.12	0.000045689	0.2874	3	153484201	153484500
EPHA6	-1.04	0.000045747	0.2874	3	97424101	97424400
DENND5B	0.40	0.000045833	0.2874	12	31514701	31515000
ENPEP	1.02	0.000045843	0.2874	4	111370801	111371100
NRXN1	-1.04	0.000045892	0.2874	2	51196201	51196500
ZIC3	-0.66	0.000046043	0.2878	X	136995901	136996200
ACVR1C	0.99	0.000046353	0.2891	2	158526601	158526900
KRT16P1	-0.85	0.000046553	0.2892	17	18336901	18337200
APLF	1.15	0.000046558	0.2892	2	68741101	68741400
CD1D	-1.13	0.000046702	0.2895	1	158157001	158157300
ADRB1	0.33	0.000046864	0.2899	10	115764301	115764600
PLS3	-0.86	0.000047215	0.2912	X	115014601	115014900

AK8	-0.68	0.000047268	0.2912	9	135715201	135715500
ZNF346	0.40	0.000047516	0.2915	5	176454001	176454300
CNIH4	0.58	0.000047520	0.2915	1	224547601	224547900
DNAH6	-0.53	0.000047729	0.2920	2	84889201	84889500
SHC3	0.73	0.000047887	0.2920	9	91711801	91712100
ZNF667	-0.90	0.000047898	0.2920	19	56950801	56951100
ZNF45	0.34	0.000047992	0.2920	19	44424901	44425200
ERG	0.73	0.000048352	0.2926	21	39735001	39735300
CTD-2258A20.5	-0.76	0.000048480	0.2926	16	66507601	66507900
UNC5D	-1.04	0.000048486	0.2926	8	35809201	35809500
IL19	-0.74	0.000048573	0.2926	1	207020401	207020700
SEZ6	-0.67	0.000048579	0.2926	17	27300901	27301200
GTF2IRD1	-0.36	0.000048824	0.2935	7	73962301	73962600
OGFRL1	0.75	0.000049140	0.2948	6	71906401	71906700
FBXL7	0.30	0.000049347	0.2950	5	15937201	15937500
MYCN	0.62	0.000049364	0.2950	2	16260001	16260300
GRIK3	-0.83	0.000049622	0.2955	1	37297801	37298100
LINC00701	0.94	0.000049647	0.2955	10	2732401	2732700
ZFP69B	0.68	0.000049765	0.2955	1	40927501	40927800
RAPGEF4	0.76	0.000050145	0.2955	2	173851501	173851800
PDSS2	0.75	0.000050215	0.2955	6	107474701	107475000
ID4	-0.99	0.000050326	0.2955	6	19353601	19353900
MRGPRF	0.68	0.000050406	0.2955	11	68763001	68763300
SMOC1	-0.57	0.000050450	0.2955	14	70306201	70306500
USP42	0.59	0.000050617	0.2955	7	6137401	6137700
CACNA1G	-0.56	0.000050898	0.2955	17	48648001	48648300
FAM118A	0.73	0.000050954	0.2955	22	45733501	45733800
DDR2	0.65	0.000050977	0.2955	1	162627301	162627600
SCRT2	-0.77	0.000051006	0.2955	20	681601	681900
MIR100HG	-0.70	0.000051095	0.2955	11	122073901	122074200
SGCD	-0.94	0.000051145	0.2955	5	156124501	156124800
SCYL3	0.89	0.000051157	0.2955	1	169824901	169825200
TIAM2	-0.93	0.000051261	0.2955	6	155436001	155436300
FBXO32	0.56	0.000051265	0.2955	8	124542901	124543200
GSTA4	-0.82	0.000051319	0.2955	6	52809901	52810200
CHAC1	0.42	0.000051527	0.2956	15	41250901	41251200
TEX15	0.72	0.000051861	0.2956	8	30688801	30689100
ATP4A	0.79	0.000051973	0.2956	19	36071701	36072000
CASC17	0.82	0.000052048	0.2956	17	69140701	69141000
ZNF585B	-0.89	0.000052246	0.2956	19	37669801	37670100
COL23A1	0.55	0.000052325	0.2956	5	177915301	177915600
EDC3	0.35	0.000052405	0.2956	15	74944501	74944800
PCED1B	-0.86	0.000052416	0.2956	12	47718601	47718900
CT64	-0.81	0.000052472	0.2956	3	162383101	162383400
CT64	1.01	0.000052512	0.2956	3	163671901	163672200
CDK3	-0.47	0.000052615	0.2956	17	73998301	73998600
ANKS1B	-0.79	0.000052640	0.2956	12	100354801	100355100
ZNF534	-0.70	0.000052689	0.2956	19	52948501	52948800
KL	0.77	0.000052710	0.2956	13	33560401	33560700
MGMT	-0.83	0.000053412	0.2989	10	131045401	131045700
RSPO2	0.67	0.000053536	0.2991	8	109098901	109099200
SNRPA	0.51	0.000053945	0.3008	19	41272801	41273100
LINC00963	0.36	0.000054470	0.3029	9	132262501	132262800
SEC14L1	0.64	0.000054531	0.3029	17	75015601	75015900
SERINC2	-0.89	0.000054807	0.3039	1	31926601	31926900
KRT7	-0.66	0.000055063	0.3040	12	52626001	52626300
ZNF208	0.94	0.000055066	0.3040	19	22177501	22177800
REG3G	-0.86	0.000055117	0.3040	2	79206301	79206600
ZNF626	-0.83	0.000055295	0.3043	19	20893801	20894100
AQP2	-0.68	0.000055419	0.3043	12	50335801	50336100
SAPCD2	0.72	0.000055641	0.3043	9	139966201	139966500
SPATA31A3	-0.70	0.000055689	0.3043	9	40348801	40349100
B4GALNT3	-0.48	0.000055931	0.3043	12	669001	669300
CDHR2	0.58	0.000055993	0.3043	5	176014201	176014500
LINC00879	-0.98	0.000055995	0.3043	3	94845001	94845300
USP17L25	-0.49	0.000055999	0.3043	4	9352201	9352500
ATRX	-0.53	0.000056231	0.3050	X	77052601	77052900
MRPL15	-0.90	0.000056477	0.3058	8	55119301	55119600
NAMPT	-0.91	0.000056891	0.3072	7	105876601	105876900
PFKP	-0.98	0.000057034	0.3072	10	3075301	3075600

GRID2	-1.06	0.000057044	0.3072	4	92967301	92967600
RIMBP2	-0.47	0.000057781	0.3103	12	131075701	131076000
IMPG2	-0.95	0.000057812	0.3103	3	100926601	100926900
NR3C2	0.59	0.000057929	0.3103	4	149109901	149110200
MIR138-1	-1.00	0.000058026	0.3103	3	44203801	44204100
C20orf26	0.84	0.000058573	0.3127	20	20073901	20074200
ARL6IP6	-1.01	0.000058704	0.3128	2	153594901	153595200
C1orf189	0.68	0.000058821	0.3129	1	154168801	154169100
KIAA1257	0.40	0.000059028	0.3134	3	128709301	128709600
XPNPEP2	-0.76	0.000059201	0.3138	X	128831701	128832000
KIAA0556	-0.67	0.000059457	0.3146	16	27776401	27776700
CLPSL2	-0.69	0.000059763	0.3151	6	35745601	35745900
SLC6A16	0.40	0.000059769	0.3151	19	49811401	49811700
DIRC3	0.67	0.000060060	0.3161	2	217940701	217941000
KCNA3	-0.97	0.000060415	0.3173	1	111260401	111260700
ZMYND8	0.64	0.000060589	0.3173	20	45958201	45958500
SPATA31A2	-0.67	0.000060608	0.3173	9	39865201	39865500
FLJ33534	-0.93	0.000060943	0.3185	2	11213101	11213400
USP17L21	-0.55	0.000061097	0.3186	4	9224401	9224700
B4GALNT2	0.82	0.000061167	0.3186	17	47216401	47216700
AC072062.1	-0.86	0.000061553	0.3200	2	215710201	215710500
YIPF3	-0.84	0.000061979	0.3207	6	43483201	43483500
CSNK1A1L	0.78	0.000061985	0.3207	13	37854901	37855200
STPG1	-0.96	0.000061996	0.3207	1	24699601	24699900
ADAMTS2	0.43	0.000062281	0.3210	5	178684201	178684500
RPL7	-0.74	0.000062375	0.3210	8	74188501	74188800
RP11-115J16.1	-0.82	0.000062617	0.3210	8	9125101	9125400
AGAP1	-0.73	0.000062619	0.3210	2	236216401	236216700
RNPEPL1	-1.51	0.000062646	0.3210	2	241518901	241519200
CADM1	-0.70	0.000062981	0.3210	11	115033201	115033500
MAP3K8	0.59	0.000063337	0.3210	10	30702901	30703200
UTRN	0.87	0.000063390	0.3210	6	144668701	144669000
MKI67	0.71	0.000063392	0.3210	10	130502701	130503000
RPS6KA2	0.34	0.000063508	0.3210	6	166950001	166950300
FHIT	0.80	0.000063537	0.3210	3	60487201	60487500
SGK1	0.68	0.000063600	0.3210	6	134595301	134595600
NRN1L	0.61	0.000063718	0.3210	16	67920901	67921200
TMCO4	-1.03	0.000063889	0.3210	1	20090101	20090400
B3GALT1	-1.16	0.000063919	0.3210	2	168646801	168647100
POTEM	0.77	0.000063952	0.3210	14	19947901	19948200
FAM118A	0.64	0.000064094	0.3210	22	45724501	45724800
ZC3H18	0.50	0.000064201	0.3210	16	88626001	88626300
DYRK1A	-0.69	0.000064256	0.3210	21	38754001	38754300
TFAP2C	-0.75	0.000064362	0.3210	20	55452901	55453200
RNPEPL1	-0.93	0.000064442	0.3210	2	241519201	241519500
TFAP2A	-0.96	0.000064457	0.3210	6	9758401	9758700
CRYGN	-0.65	0.000064519	0.3210	7	151149301	151149600
SLC13A4	-0.88	0.000064642	0.3211	7	135398401	135398700
PKNOX2	-0.70	0.000064983	0.3219	11	125121901	125122200
MTA1	-0.65	0.000065030	0.3219	14	105921901	105922200
LOC102031319	0.71	0.000065274	0.3222	10	32647501	32647800
CACNA2D2	-0.84	0.000065306	0.3222	3	50460001	50460300
ERC2	-0.73	0.000065979	0.3247	3	56298601	56298900
TMEM181	-0.82	0.000066025	0.3247	6	158969401	158969700
GABRG1	-0.91	0.000066282	0.3254	4	45763501	45763800
GHITM	0.63	0.000066421	0.3254	10	85829401	85829700
FMNL2	0.90	0.000066483	0.3254	2	153122101	153122400
SNX5	-0.63	0.000066874	0.3267	20	17900401	17900700
TCERG1L	-0.40	0.000067030	0.3270	10	132869401	132869700
ZMIZ1-AS1	-1.11	0.000067221	0.3271	10	80761201	80761500
FIP1L1	1.08	0.000067608	0.3271	4	54250201	54250500
CSNK1G1	-0.42	0.000067619	0.3271	15	64611601	64611900
PNLDC1	0.51	0.000067807	0.3271	6	160248901	160249200
ZCCHC7	-0.97	0.000067910	0.3271	9	37228801	37229100
HDGFL1	-0.89	0.000067958	0.3271	6	23348101	23348400
MIR30D	0.91	0.000067984	0.3271	8	135981601	135981900
LINC00708	-0.90	0.000068040	0.3271	10	8727001	8727300
LOC729737	-1.21	0.000068101	0.3271	1	132901	133200
NPAS2	-0.87	0.000068404	0.3271	2	101441401	101441700
HNRNPA0	-0.86	0.000068443	0.3271	5	137109301	137109600

LIFR-AS1	-0.93	0.000068765	0.3271	5	38660401	38660700
RNGTT	-1.11	0.000068926	0.3271	6	89309401	89309700
OTUD1	-0.62	0.000069153	0.3271	10	23810401	23810700
LINC00605	-0.74	0.000069191	0.3271	14	103631401	103631700
BAI1	-0.90	0.000069210	0.3271	8	143540101	143540400
AJAP1	-0.84	0.000069210	0.3271	1	4773601	4773900
MAPRE3	-0.99	0.000069247	0.3271	2	27244501	27244800
CITED2	-0.70	0.000069284	0.3271	6	139718401	139718700
DCTN1	0.45	0.000069446	0.3271	2	74582401	74582700
RP11-536C10.7	0.82	0.000069608	0.3271	14	19396801	19397100
RABEP1	0.29	0.000069696	0.3271	17	5173501	5173800
NXNL2	0.62	0.000069848	0.3271	9	91146301	91146600
NXN	-0.52	0.000069859	0.3271	17	781501	781800
TNS3	-0.96	0.000070087	0.3271	7	47536501	47536800
PRR16	0.85	0.000070096	0.3271	5	119397601	119397900
MIR6085	-0.73	0.000070112	0.3271	15	62759401	62759700
IQGAP2	-0.92	0.000070302	0.3271	5	75784501	75784800
SDHAP3	-0.97	0.000070312	0.3271	5	1580101	1580400
FLJ12825	0.85	0.000070327	0.3271	12	54460201	54460500
TPD52	0.87	0.000070468	0.3271	8	81117301	81117600
GMDS	0.75	0.000070644	0.3271	6	2229601	2229900
SRGAP3	-0.96	0.000070653	0.3271	3	9033301	9033600
CHRM3	-1.07	0.000070764	0.3271	1	239422801	239423100
C6orf48	-1.22	0.000071008	0.3275	6	31811701	31812000
FGF1	-0.95	0.000071121	0.3275	5	141838201	141838500
TTC8	1.01	0.000071179	0.3275	14	89433601	89433900
VSTM4	-0.78	0.000071327	0.3276	10	50208601	50208900
PURB	0.42	0.000071501	0.3276	7	44956201	44956500
PLXNA4	0.77	0.000071535	0.3276	7	132164401	132164700
ARHGEF26-AS1	-0.50	0.000071675	0.3278	3	153515401	153515700
SNX30	-0.71	0.000072126	0.3284	9	115569601	115569900
LINC00669	-0.58	0.000072205	0.3284	18	37257901	37258200
LINC00398	-0.90	0.000072226	0.3284	13	31393501	31393800
CPNE5	-0.65	0.000072256	0.3284	6	36754801	36755100
DLGAP4	-0.65	0.000072404	0.3285	20	35022601	35022900
SPP1	-0.85	0.000072486	0.3285	4	88862101	88862400
CDK19	-0.89	0.000073173	0.3301	6	111001801	111002100
CTPS2	0.62	0.000073209	0.3301	X	16733101	16733400
ERCC4	-0.81	0.000073277	0.3301	16	14097301	14097600
PET112	0.60	0.000073292	0.3301	4	152847901	152848200
ZCCHC7	-0.74	0.000073671	0.3313	9	37371301	37371600
LPHN2	-1.09	0.000073837	0.3316	1	83242801	83243100
DLG2	-0.96	0.000074060	0.3320	11	83887801	83888100
SLC39A11	0.68	0.000074493	0.3320	17	70698001	70698300
LINC00645	-0.97	0.000074643	0.3320	14	28636501	28636800
EML5	-0.82	0.000074705	0.3320	14	89110201	89110500
OLFM1	-0.99	0.000074728	0.3320	9	137965801	137966100
LOC284344	-1.01	0.000074850	0.3320	19	43751401	43751700
GPT2	-0.49	0.000074971	0.3320	16	46936201	46936500
CDC42EP3	0.78	0.000074974	0.3320	2	37953001	37953300
NMBR	0.62	0.000075016	0.3320	6	142157101	142157400
LOC101927881	0.46	0.000075116	0.3320	2	129793201	129793500
TDRD3	-1.07	0.000075141	0.3320	13	60999601	60999900
IL13RA1	0.60	0.000075350	0.3320	X	117846601	117846900
MTBP	0.91	0.000075444	0.3320	8	121481101	121481400
LINC01140	0.45	0.000075477	0.3320	1	87707701	87708000
FAM26F	-0.81	0.000075620	0.3321	6	116775001	116775300
COL5A1	0.35	0.000075956	0.3325	9	137439001	137439300
COL6A2	-0.97	0.000076127	0.3325	21	47481901	47482200
AL109763.2	-0.74	0.000076200	0.3325	21	20535901	20536200
TBL1X	-0.59	0.000076256	0.3325	X	9515401	9515700
IFITM3	-0.90	0.000076786	0.3325	11	336301	336600
GLIS1	-0.94	0.000076796	0.3325	1	54155401	54155700
KNTC1	0.64	0.000077202	0.3325	12	123108901	123109200
PABPC4L	-0.57	0.000077379	0.3325	4	135548401	135548700
NELL1	-0.96	0.000077400	0.3325	11	21396301	21396600
LINC00702	-0.59	0.000077437	0.3325	10	4198501	4198800
ZNF516	-0.49	0.000077526	0.3325	18	74067001	74067300
RP11-138B4.1	0.90	0.000077566	0.3325	4	188529901	188530200
RP3-513G18.2	-0.99	0.000077618	0.3325	4	3701701	3702000

MCOLN3	0.70	0.000077694	0.3325	1	85520401	85520700
FHL2	-0.87	0.000077723	0.3325	2	106082701	106083000
SNRPN	-0.53	0.000077749	0.3325	15	25176601	25176900
PACRG	0.85	0.000077752	0.3325	6	163361701	163362000
CDKL2	-1.07	0.000077843	0.3325	4	76520701	76521000
METTL16	0.71	0.000077912	0.3325	17	2404801	2405100
TMEM184C	-1.02	0.000077924	0.3325	4	148520701	148521000
GCFC2	0.91	0.000078348	0.3339	2	76337701	76338000
BRINP1	0.63	0.000078635	0.3340	9	121946101	121946400
ACTL8	0.59	0.000078649	0.3340	1	18073501	18073800
SCFD2	-0.93	0.000078720	0.3340	4	53959201	53959500
MIR548BA	-0.59	0.000079179	0.3344	5	2014501	2014800
TICRR	0.74	0.000079294	0.3344	15	90104101	90104400
UGT2A1	-1.09	0.000079528	0.3344	4	70462201	70462500
ARL6IP6	-1.30	0.000079609	0.3344	2	153777601	153777900
DAG1	0.51	0.000079714	0.3344	3	49515301	49515600
STARD8	-0.68	0.000079788	0.3344	X	67895101	67895400
CD99P1	-0.81	0.000079950	0.3344	X	2586301	2586600
RP1-60O19.1	-0.79	0.000080080	0.3344	6	107289301	107289600
NDUFB11	1.07	0.000080089	0.3344	X	46987501	46987800
GTF3C1	0.72	0.000080092	0.3344	16	27505201	27505500
PPARD	0.39	0.000080125	0.3344	6	35325301	35325600
CD320	-0.66	0.000080150	0.3344	19	8358901	8359200
RP11-421P23.1	0.80	0.000080302	0.3346	8	20542501	20542800
DTWD2	-0.75	0.000080553	0.3349	5	118345201	118345500
C1orf21	-0.75	0.000080601	0.3349	1	184535401	184535700
CCNG2	0.87	0.000080808	0.3353	4	78040201	78040500
MIR202HG	-0.40	0.000081105	0.3357	10	135061501	135061800
STK3	0.50	0.000081131	0.3357	8	99804601	99804900
DNAH5	0.80	0.000081408	0.3364	5	13861801	13862100
HNRNPD	-0.65	0.000081524	0.3364	4	83283601	83283900
ASAH2B	0.41	0.000081795	0.3371	10	52487701	52488000
BOLA3	0.43	0.000082087	0.3376	2	74358001	74358300
MIR548AJ2	-0.95	0.000082150	0.3376	4	23174401	23174700
PRPF4B	0.59	0.000082333	0.3379	6	3966901	3967200
ZNF584	0.55	0.000082577	0.3380	19	58923301	58923600
NR2C1	0.67	0.000082704	0.3380	12	95431501	95431800
RHOB	-0.90	0.000082756	0.3380	2	20672701	20673000
PAX4	-0.80	0.000082827	0.3380	7	127251001	127251300
HGFAC	-0.77	0.000082997	0.3380	4	3453001	3453300
MIR5696	-0.97	0.000083079	0.3380	2	101941201	101941500
EFCAB6	-0.46	0.000083162	0.3380	22	43938901	43939200
UNC5C	-0.76	0.000083393	0.3380	4	96190201	96190500
RBBP7	0.94	0.000083441	0.3380	X	16917601	16917900
CSMD1	0.75	0.000083601	0.3380	8	4131601	4131900
GRK4	0.74	0.000083700	0.3380	4	3002101	3002400
FAM19A5	-0.58	0.000083707	0.3380	22	48999601	48999900
SLC2A3	0.63	0.000083841	0.3381	12	8126101	8126400
PTPN9	-0.61	0.000084030	0.3382	15	75794701	75795000
CRLF2	-0.52	0.000084096	0.3382	Y	1294501	1294800
DHRS3	0.51	0.000084243	0.3383	1	12633001	12633300
GFPT1	0.63	0.000084771	0.3400	2	69563101	69563400
CEP112	1.10	0.000085065	0.3404	17	63961501	63961800
LOC643441	1.02	0.000085110	0.3404	1	113804401	113804700
SDK1	0.36	0.000085669	0.3421	7	4342501	4342800
PDE4A	-0.75	0.000085848	0.3421	19	10547701	10548000
BLCAP	0.95	0.000085987	0.3421	20	36096001	36096300
ROBO1	-1.09	0.000086083	0.3421	3	78450901	78451200
TBC1D3P1-DHX40	-0.63	0.000086245	0.3421	17	58052101	58052400
GPHN	-0.66	0.000086413	0.3421	14	67604101	67604400
RP5-1166F10.1	-0.68	0.000086428	0.3421	1	4388701	4389000
MYOM3	-0.69	0.000086513	0.3421	1	24399301	24399600
ONECUT1	0.65	0.000086553	0.3421	15	53409301	53409600
CT64	0.57	0.000086910	0.3431	3	163219501	163219800
LRRCC1	0.94	0.000087282	0.3437	8	86060401	86060700
POC5	0.35	0.000087302	0.3437	5	75141601	75141900
TRIM60	-0.87	0.000087871	0.3441	4	165966601	165966900
APOH	-0.71	0.000088117	0.3441	17	64207801	64208100
TCERG1L	-0.43	0.000088295	0.3441	10	132869101	132869400
SLITRK1	-0.61	0.000088507	0.3441	13	82738201	82738500

C10orf90	-0.53	0.000088731	0.3441	10	128151001	128151300
PRDM9	-0.71	0.000088742	0.3441	5	23997601	23997900
LOC100287015	1.02	0.000088767	0.3441	8	5844601	5844900
NUCKS1	1.10	0.000088952	0.3441	1	205695901	205696200
PTPN22	0.85	0.000089018	0.3441	1	114395401	114395700
NFE2	1.07	0.000089172	0.3441	12	54695401	54695700
RTF1	0.76	0.000089941	0.3441	15	41703901	41704200
STK33	-0.73	0.000090099	0.3441	11	8404201	8404500
KIAA1432	-0.87	0.000090148	0.3441	9	5606701	5607000
CD109	-1.08	0.000090190	0.3441	6	74892901	74893200
LRRC59	0.95	0.000090310	0.3441	17	48481201	48481500
CACNA1E	-1.13	0.000090707	0.3441	1	181405801	181406100
OCA2	0.85	0.000090722	0.3441	15	28286101	28286400
TRAPPC11	1.08	0.000090727	0.3441	4	184673401	184673700
TTLL4	0.89	0.000090899	0.3441	2	219624901	219625200
WNT1	-0.84	0.000090946	0.3441	12	49375501	49375800
ZNF608	-0.92	0.000090990	0.3441	5	124031101	124031400
SPATA31A3	-0.75	0.000091029	0.3441	9	40675501	40675800
EYS	0.84	0.000091166	0.3441	6	65384401	65384700
BUD13	-0.89	0.000091249	0.3441	11	116226001	116226300
LOC100506470	-1.02	0.000091278	0.3441	20	59676601	59676900
LEO1	0.70	0.000091321	0.3441	15	52225801	52226100
CENPC	-0.92	0.000091407	0.3441	4	68184001	68184300
AXIN2	0.46	0.000091481	0.3441	17	63541501	63541800
DGKB	-1.03	0.000091596	0.3441	7	14157601	14157900
LRRFIP1	-0.53	0.000091682	0.3441	2	238683901	238684200
SAMD4A	-0.77	0.000091876	0.3441	14	55089601	55089900
CBL	-0.61	0.000091886	0.3441	11	119143801	119144100
MIR205HG	0.79	0.000091903	0.3441	1	209184901	209185200
EHMT1	-0.81	0.000092062	0.3441	9	140604601	140604900
AP000469.2	-1.01	0.000092124	0.3441	21	25439101	25439400
SYT4	0.41	0.000092161	0.3441	18	41368201	41368500
INPP1	-1.08	0.000092314	0.3441	2	191243701	191244000
RP11-140I24.1	0.61	0.000092357	0.3441	16	73622401	73622700
UNC5D	-1.05	0.000092527	0.3441	8	34387801	34388100
ZNF707	0.62	0.000092559	0.3441	8	144755701	144756000
CXorf57	-0.87	0.000092631	0.3441	X	105736801	105737100
RP1-60O19.1	-0.53	0.000092646	0.3441	6	107163001	107163300
POLR3B	0.50	0.000092678	0.3441	12	106746001	106746300
DSEL	0.70	0.000092745	0.3441	18	65175601	65175900
XXYLT1	-0.46	0.000092772	0.3441	3	194964901	194965200
C3orf18	-0.94	0.000092788	0.3441	3	50591701	50592000
AGR2	0.78	0.000092806	0.3441	7	16869901	16870200
EGFL6	-0.83	0.000092908	0.3441	X	13518901	13519200
MAOA	-1.05	0.000093111	0.3441	X	43317901	43318200
AKAP6	0.67	0.000093207	0.3441	14	32787601	32787900
TTC34	-0.35	0.000093258	0.3441	1	2700301	2700600
ZFAND5	0.65	0.000093520	0.3444	9	74990101	74990400
GRM7	-0.80	0.000093665	0.3444	3	7425901	7426200
SLC38A4	0.48	0.000093670	0.3444	12	47218201	47218500
CNPY1	-0.46	0.000093886	0.3444	7	155307301	155307600
N6AMT1	0.88	0.000093915	0.3444	21	30141601	30141900
LINC01139	1.43	0.000094023	0.3444	1	238719001	238719300
ABHD6	-0.71	0.000094397	0.3454	3	58242301	58242600
	-0.84	0.000094979	0.3459	Un_gl000246	16801	17100
GRIK3	-0.95	0.000095071	0.3459	1	37278601	37278900
ABCB4	-0.72	0.000095135	0.3459	7	87035401	87035700
COL18A1	-0.66	0.000095137	0.3459	21	46915801	46916100
UBXN7	0.68	0.000095144	0.3459	3	196132201	196132500
ATXN1L	0.75	0.000095307	0.3459	16	71891701	71892000
ITGAM	1.03	0.000095361	0.3459	16	31302301	31302600
TADA1	0.68	0.000095511	0.3460	1	166842301	166842600
KIF13B	-0.70	0.000095662	0.3460	8	28917601	28917900
LINC00900	0.88	0.000095802	0.3460	11	115881601	115881900
CKMT2-AS1	0.82	0.000095907	0.3460	5	80546701	80547000
DHRS3	0.44	0.000095958	0.3460	1	12633301	12633600
NAP1L4	-0.88	0.000096729	0.3484	11	2981401	2981700
BUD13	-0.63	0.000096938	0.3487	11	116428801	116429100
TSHZ2	0.75	0.000097226	0.3493	20	51832201	51832500
AC010987.6	-1.05	0.000097691	0.3496	2	68060401	68060700

SIDT1	0.94	0.000097792	0.3496	3	113329201	113329500
RP5-1086L22.1	-0.34	0.000097960	0.3496	6	170552101	170552400
RFPL4B	-0.72	0.000097973	0.3496	6	112670701	112671000
RP11-1134I14.8	-1.03	0.000098253	0.3496	8	48095101	48095400
VWA8	0.47	0.000098254	0.3496	13	42534001	42534300
LOC401324	-1.01	0.000098271	0.3496	7	35488201	35488500
ZNF565	-0.70	0.000098291	0.3496	19	36692401	36692700
LOC100506746	-0.84	0.000098434	0.3496	4	87838801	87839100
CTNNBL1	0.89	0.000098568	0.3496	20	36372901	36373200
CYP4F2	0.37	0.000098586	0.3496	19	15989401	15989700
SNRPC	-0.84	0.000098866	0.3501	6	34736101	34736400
YES1	0.67	0.000098953	0.3501	18	815101	815400
RAB37	0.71	0.000099247	0.3504	17	72722101	72722400
PTPRF	-0.66	0.000099287	0.3504	1	44080801	44081100
ASPG	0.58	0.000099530	0.3509	14	104537701	104538000
ABTB2	0.45	0.000099823	0.3515	11	34179901	34180200

Table S4. A selection of the top results from the functional annotation analysis that provides more evidence that Hi cortisol affect methylation near genes associated with nervous system and calcium transport, as well as hypomethylation.

Category	*Association*	*Genes*	*Database*	*Identifyer*	*Statistics*
Nervous system					
	Nervous system development	BDH1; ABLIM2; ACSL3; ADAM22; ATRX; ALMS1; APOD; ATG7; BAI1; CDH4; CDH23; CACNB2; CACNA1S; CACNA1G; CACNA1I; CTNNA1; CITED2; CADM1; CHAC1; CHRM3; COL4A1; COL5A1; COL6A2; CDK1; DLG2; DYRK1A; DCTN1; DAG1; FGF1; GFRA1; GRIN3A; GPM6A; ID4; INSC; IGF1R; ITM2B; ITGAM; INTU; KATNB1; KLHL1; KIF13B; LPHN2; MESP1; MCOLN3; NELL1; NGF; NRXN1; NRN1L; NPAS2; NRP2; NAIP; NCOA1; OLFM1; OTX2; OGDH; PHOX2B; PCM1; PPARD; PIP5K1C; PLXNA4; PLXNC1; PRDM6; PRNP; PICK1; PTPRD; PTPRF; RAPGEF4; RHOB; RGS14; RTN4; RXRA; ARHGEF10; RPS6KA2; RNF6; ROBO1; SFRP1; SPP1; SEZ6; SEMA4D; SEMA5A; SEMA7A; SERINC5; STK3; SHC3; SLITRK1; SMAD9; SCN8A; STRN; SOD2; THBS2; TCF7; TFAP2A; TFAP2C; TULP1; TP53BP2; UNC5C; UNC5D; MYCN; WNT1; ZIC3; ZBTB16	GO biological process	GO:0007399	C=1724; O=101; E=62.46; R=1.62; rawP=5.22e-07; adjP=0.0005
	Neuron development	ABLIM2; ALMS1; APOD; ATG7; BAI1; CDH4; CDH23; CACNB2; CACNA1S; CACNA1G; CACNA1I; CTNNA1; COL4A1; COL5A1; COL6A2; CDK1; DLG2; GFRA1; GRIN3A; GPM6A; IGF1R; KATNB1; KLHL1; KIF13B; NGF; NRXN1; NRP2; OTX2; OGDH; PHOX2B; PIP5K1C; PLXNA4; PLXNC1; PRNP; PICK1; PTPRD; PTPRF; RAPGEF4; RHOB; RTN4; RXRA; RPS6KA2; RNF6; ROBO1; SPP1; SEZ6; SEMA4D; SEMA5A; SEMA7A; SLITRK1; SCN8A; STRN; SOD2; TFAP2A; TULP1; UNC5C; UNC5D	GO biological process	GO:0048666	C=798; O=57; E=28.91; R=1.97; rawP=6.69e-07; adjP=0.0005
	Neurogenesis	ABLIM2; ADAM22; ALMS1; APOD; ATG7; BAI1; CDH4; CDH23; CACNB2; CACNA1S; CACNA1G; CACNA1I; CTNNA1; CHAC1; COL4A1; COL5A1; COL6A2; CDK1; DLG2; DAG1; GFRA1; GRIN3A; GPM6A; ID4; IGF1R; ITGAM; KATNB1; KLHL1; KIF13B; MESP1; MCOLN3; NGF; NRXN1; NRP2; NCOA1; OTX2; OGDH; PHOX2B; PCM1; PIP5K1C; PLXNA4; PLXNC1; PRDM6; PRNP; PICK1; PTPRD; PTPRF; RAPGEF4; RHOB; RGS14; RTN4; RXRA; ARHGEF10; RPS6KA2; RNF6; ROBO1; SPP1; SEZ6; SEMA4D; SEMA5A; SEMA7A; SLITRK1; SCN8A; STRN; SOD2; TCF7; TFAP2A; TFAP2C; TULP1; UNC5C; UNC5D; MYCN; WNT1	GO biological process	GO:0022008	C=1140; O=73; E=41.30; R=1.77; rawP=1.12e-06; adjP=0.0007
	Neuron projection development	ABLIM2; APOD; ATG7; BAI1; CDH4; CACNB2; CACNA1S; CACNA1G; CACNA1I; CTNNA1; COL4A1; COL5A1; COL6A2; CDK1; GFRA1; GRIN3A; GPM6A; IGF1R; KATNB1; KLHL1; KIF13B; NGF; NRXN1; NRP2; OTX2; PHOX2B; PIP5K1C; PLXNA4; PLXNC1; PRNP; PTPRD; PTPRF; RAPGEF4; RHOB; RTN4; RXRA; RPS6KA2; RNF6; ROBO1; SPP1; SEZ6; SEMA4D; SEMA5A; SEMA7A; SLITRK1; SCN8A; STRN; TULP1; UNC5C; UNC5D	GO biological process	GO:0031175	C=704; O=50; E=25.50; R=1.96; rawP=3.99e-06; adjP=0.0014
	Neuron projection morphogenesis	ABLIM2; APOD; ATG7; BAI1; CDH4; CACNB2; CACNA1S; CACNA1G; CACNA1I; CTNNA1; COL4A1; COL5A1; COL6A2; CDK1; GFRA1; GPM6A; IGF1R; KIF13B; NGF; NRXN1; NRP2; OTX2; PHOX2B; PIP5K1C; PLXNA4; PLXNC1; PRNP; PTPRD; PTPRF; RHOB; RTN4; RXRA; RPS6KA2; RNF6; ROBO1; SPP1; SEMA4D; SEMA5A; SEMA7A; SLITRK1; SCN8A; UNC5C; UNC5D	GO biological process	GO:0048812	C=583; O=43; E=21.12; R=2.04; rawP=7.71e-06; adjP=0.0016
	Cell morphogenesis involved in neuron differentiation	ABLIM2; APOD; ATG7; BAI1; CDH4; CDH23; CACNB2; CACNA1S; CACNA1G; CACNA1I; CTNNA1; COL4A1; COL5A1; COL6A2; CDK1; GFRA1; IGF1R; KIF13B; NGF; NRXN1; NRP2; OTX2; PHOX2B; PIP5K1C; PLXNA4; PLXNC1; PRNP; PTPRD; PTPRF; RHOB; RTN4; RXRA; RPS6KA2; RNF6; ROBO1; SPP1; SEMA4D; SEMA5A; SEMA7A; SLITRK1; SCN8A; UNC5C; UNC5D	GO biological process	GO:0048667	C=575; O=43; E=20.83; R=2.06; rawP=5.45e-06; adjP=0.0016

Neuron projection	ADCYAP1R1; APBB2; ANKS1B; APOD; ATXN1L; AXIN2; CTNND2; CADM1; CHRM3; CHRNA4; CPNE5; DENND1A; DLGAP4; DLG2; DAG1; ERC2; GRK4; GABBR2; GRID2; GRIK3; GRIN3A; GRM7; GPM6A; GNB2L1; IGF1R; KATNB1; KLHL1; KIF13B; MME; NRXN1; NRP2; OTX2; PICK1; PTPRF; RAPGEF4; RGS14; RPTOR; RTN4; RXRA; RNF6; ROBO1; SEZ6; SHANK2; SUMO1; SCN8A; SAMD4A; STRN; SYT4; SNCG; TIAM2; TPGS1	GO cellular component	GO:0043005	C=651; O=51; E=22.68; R=2.25; rawP=5.54e-08; adjP=1.86e-05
Dendrite	ANKS1B; APOD; ATXN1L; AXIN2; CTNND2; CADM1; CHRM3; CHRNA4; DENND1A; DLGAP4; DLG2; GRK4; GRID2; GRIK3; GRIN3A; GRM7; GPM6A; GNB2L1; KLHL1; MME; PICK1; RAPGEF4; RGS14; RPTOR; SEZ6; SHANK2; SUMO1; SAMD4A; STRN; TPGS1	GO cellular component	GO:0030425	C=355; O=30; E=12.37; R=2.43; rawP=7.48e-06; adjP=0.0008
Synaptic membrane	ANKS1B; CHRM3; CHRNA4; DENND1A; DLG2; DAG1; ERC2; GABRG1; GABBR2; GPHN; GRID2; GRIK3; GRIN3A; GRM7; NRXN1; PICK1; RIMS1; RGS14; SHANK2; STRN; UTRN	GO cellular component	GO:0097060	C=215; O=21; E=7.49; R=2.80; rawP=2.11e-05; adjP=0.0012
Synapse	APBB2; ANKS1B; AXIN2; CDH23; CTNND2; CADM1; CHRM3; CHRNA4; DENND1A; DLGAP4; DLG2; DAG1; ERC2; GABRG1; GABBR2; GPHN; GRID2; GRIK3; GRIN3A; GRM7; MME; NRXN1; PICK1; PTPRF; RAPGEF4; RIMS1; RGS14; RIMBP2; SHANK2; SUMO1; SAMD4A; STRN; SYT4; SYTL4; TULP1; UTRN	GO cellular component	GO:0045202	C=489; O=36; E=17.03; R=2.11; rawP=2.06e-05; adjP=0.0012
Synapse part	ANKS1B; AXIN2; CTNND2; CADM1; CHRM3; CHRNA4; DENND1A; DLG2; DAG1; ERC2; GABRG1; GABBR2; GPHN; GRID2; GRIK3; GRIN3A; GRM7; MME; NRXN1; PICK1; RIMS1; RGS14; SHANK2; STRN; SYT4; SYTL4; UTRN	GO cellular component	GO:0044456	C=370; O=27; E=12.89; R=2.09; rawP=0.0003; adjP=0.0126
Neuronal cell body	APOD; CTNND2; CHRNA4; CPNE5; DENND1A; DLGAP4; DLG2; GRK4; GRIK3; GRIN3A; GPM6A; GNB2L1; KATNB1; KLHL1; PHAX; PTPRF; RAB2A; RPTOR; RTN4; ROBO1; STRN; SNCG	GO cellular component	GO:0043025	C=291; O=22; E=10.14; R=2.17; rawP=0.0006; adjP=0.0168
Axon guidance	ABLIM2; EPHA6; PLXNC1; ROBO1; SEMA4D; SEMA5A; SEMA7A; SRGAP3; UNC5C; UNC5D	KEGG pathway	4360	C=129; O=10; E=2.17; R=4.62; rawP=6.85e-05; adjP=0.0019
Neuroactive ligand-receptor interaction	ADCYAP1R1; ADRB1; CHRM3; CHRNA4; GABRG1; GABBR2; GRID2; GRIK3; GRIN3A; GRM7; MCHR2; NMBR	KEGG pathway	4080	C=272; O=12; E=4.57; R=2.63; rawP=0.0024; adjP=0.0262
Neural Crest Differentiation	AXIN2; CDH6; PHOX2B; RHOB; TFAP2A; MYB; WNT1	Wiki pathways	WP2064	C=123; O=7; E=2.06; R=3.39; rawP=0.0049; adjP=0.0462
Axon guidance	ABLIM2; CACNB2; CACNA1S; CACNA1G; CACNA1I; CDK1; GFRA1; NGF; NRP2; PIP5K1C; PLXNA4; PLXNC1; PRNP; RHOB; RPS6KA2; ROBO1; SEMA4D; SEMA5A; SEMA7A; UNC5C; UNC5D	Pathway Commons	DB_ID:20	C=219; O=21; E=3.68; R=5.71; rawP=1.83e-10; adjP=3.29e-08
Mental Disorders	ATRX; APBB2; APOD; RNPEPL1; ARNTL; BTBD9; CTNNA3; CRBN; CHRNA4; CNTN5; DYRK1A; GABRG1; GABBR2; GRIK3; GRIN3A; GRM7; GPM6A; IMMP2L; INPP1; IDE; ITM2B; MME; MAOA; NGF; NRXN1; NPAS2; PKNOX2; PCM1; PER3; PRNP; PICK1; PTPRD; RTN4; SETBP1; SLITRK1	Disease association	DB_ID:PA447208	C=564; O=35; E=9.47; R=3.70; rawP=5.29e-11; adjP=2.32e-08
Nervous System Diseases	ADAM22; ATRX; ALOX5AP; BAI1; BCKDHB; BTBD9; CDH23; CACNA1S; CTNNA3; CHRNA4; COL4A1; COL6A2; DYRK1A; DCTN1; DAG1; FBXO32; GTF2IRD1; IDE; ITM2B; LITAF; MME; NGF; NAIP; MGMT; OTX2; PHOX2B; PACRG; PER3; PRNP; RFC1; RTN4; SGCD; SLITRK1; SNRPN; SCN8A; TTC8;	Disease association	DB_ID:PA445093	C=694; O=37; E=11.65; R=3.18; rawP=1.03e-09; adjP=2.26e-07
Bupropion (anitdepressant drug)	ATP8A1; CACNB2; CD109; CSMD1; ERC2; NELL1; PTPRD; SEMA5A; THSD4	Drug interaction	DB_ID:PA448687	C=108; O=9; E=1.81; R=4.96; rawP=9.00e-05; adjP=0.0027

Calcium transport				
Calcium ion transmembrane transporter activity	ATP2B3; CACNA2D2; CACNB2; CACNA1S; CACNA1E; CACNA1G; CACNA1I; GRIN3A; GRM7; GPM6A; MCU; SLC24A2; STIM2	GO molecular function	GO:0015085	C=113; O=13; E=3.96; R=3.29; rawP=0.0002; adjP=0.0229
Voltage-gated calcium channel activity	CACNA2D2; CACNB2; CACNA1S; CACNA1E; CACNA1G; CACNA1I; GRM7	GO molecular function	GO:0005245	C=36; O=7; E=1.26; R=5.55; rawP=0.0002; adjP=0.0229
Calcium channel activity	CACNA2D2; CACNB2; CACNA1S; CACNA1E; CACNA1G; CACNA1I; GRIN3A; GRM7; GPM6A; MCU; SLC24A2; STIM2	GO molecular function	GO:0005262	C=97; O=12; E=3.40; R=3.53; rawP=0.0001; adjP=0.0229
Calcium channel complex	AKAP6; CACNA2D2; CACNB2; CACNA1S; CACNA1E; CACNA1G; CACNA1I	GO cellular component	GO:0034704	C=41; O=7; E=1.43; R=4.90; rawP=0.0005; adjP=0.0168
Calcium Regulation in the Cardiac Cell	ADRB1; ATP2B3; CACNA1S; CACNA1E; CAMK4; CHRM3; GRK4; RGS14	Wikipathways pathway	WP536	C=151; O=8; E=2.53; R=3.16; rawP=0.0041; adjP=0.0451
Epigenetic regulation				
Decitabine (hypomethylates DNA)	CADM1; FHIT; ID4; RARRES1; ROBO1; SFRP1; SNCG; TEX15	Drug interaction	DB_ID:PA164749631	C=79; O=8; E=1.33; R=6.03; rawP=5.55e-05; adjP=0.0020
Azacitidine (hypomethylates DNA)	AXIN2; CADM1; ID4; MGMT; PAX4; PER3; RARRES1; ROBO1; SFRP1; SNCG; TEX15	Drug interaction	DB_ID:PA451996	C=103; O=11; E=1.73; R=6.36; rawP=1.36e-06; adjP=0.0002

Analysis was done using the WebGestalt functional gene annotation web tool.

Of the 852 DMR's 724 mapped to unique Entrez Gene IDs in the WebGestalt functional annotation tool.

C= number of reference genes in the category

O= number of genes in the gene set and also in the category

E= expected number in the category

R= ratio of enrichment

rawP= p value from hypergeometric test

adjp= p value adjusted by multiple test adjustment using BH method

Table S5. Primers used for bisulfite pyrosequencing

Assay	*Primers**	*5´ modification*	*Type of primer*	*Name of closest gene*
1	TATGTTTTTGTTATAGGGGAAATGATG	None	PCR Forward	BRCA1
	CCAACATAATAAAACCCCACATCT	Biotinylated	PCR Reverse	BRCA1
	TGGTATAATTTTAGTTTAGTGTAAT	None	Sequencing	BRCA1
2	AGATGGGAAGGAAGAGAAAAGA	None	PCR Forward	CACNA1S
	TACCTCCCCCTCCCACCATA	Biotinylated	PCR Reverse	CACNA1S
	AGTATGAGATGATGGTT	None	Sequencing	CACNA1S
3	TGTTGGGGTTATAGGTGTGAG	None	PCR Forward	PRDM14
	AACCTATTAATCACTTCCTAAAATATACC	Biotinylated	PCR Reverse	PRDM14
	GGTTATAGGTGTGAGT	None	Sequencing	PRDM14

* Cycling conditions for all assays: (1) Activation 15 min 95°C, (2) Cycling x45 Denaturaion 30 sec 94°C - Annealing 30 sec 56°C - Extension 30s 72°C, (3) Final extension 10 min 72°C
